# Autophagy blockade synergistically enhances nanosonosensitizer-enabled sonodynamic cancer nanotherapeutics

**DOI:** 10.1186/s12951-021-00855-y

**Published:** 2021-04-20

**Authors:** Liqiang Zhou, Minfeng Huo, Xiaoqin Qian, Li Ding, Luodan Yu, Wei Feng, Xinwu Cui, Yu Chen

**Affiliations:** 1 Sino-German Tongji-Caritas Research Center of Ultrasound in Medicine, Department of Medical Ultrasound, Tongji Hospital, Tongji Medical College, Huazhong University of Science and Technology, Wuhan, 430030 People’s Republic of China; 2 School of Life Sciences, Shanghai University, Shanghai, 200444 People’s Republic of China; 3 State Key Laboratory of High Performance Ceramics and Superfine Microstructure, Shanghai Institute of Ceramics, Chinese Academy of Sciences, Shanghai, 200050 People’s Republic of China; 4 Department of Ultrasound, Affiliated People’s Hospital of Jiangsu University, Zhenjiang, 212002 People’s Republic of China

**Keywords:** Autophagy, Sonodynamic therapy, Autophagy inhibition, Nanoliposomes, Tumor therapy

## Abstract

Ultrasound-triggered sonodynamic therapy (SDT) represents an emerging therapeutic modality for cancer treatment based on its specific feature of noninvasiveness, high tissue-penetrating depth and desirable therapeutic efficacy, but the SDT-induced pro-survival cancer-cell autophagy would significantly lower the SDT efficacy for cancer treatment. Here we propose an “all-in-one” combined tumor-therapeutic strategy by integrating nanosonosensitizers-augmented noninvasive SDT with autophagy inhibition based on the rationally constructed nanoliposomes that co-encapsulates clinically approved sonosensitizers protoporphyrin IX (PpIX) and early-phase autophagy-blocking agent 3-methyladenine (3-MA). It has been systematically demonstrated that nanosonosensitizers-augmented SDT induced cytoprotective pro-survival autophagy through activation of MAPK signaling pathway and inhibition of AMPK signaling pathway, and this could be efficaciously inhibited by 3-MA in early-phase autophagy, which significantly decreased the cell resistance to intracellular oxidative stress and complied a remarkable synergistic effect on SDT medicated cancer-cell apoptosis both in vitro at cellular level and in vivo on tumor-bearing animal model. Therefore, our results provide a proof-of-concept combinatorial tumor therapeutics based on nanosonosensitizers for the treatment of ROS-resistant cancer by autophagy inhibition-augmented SDT.

**Supplementary Information:**

The online version contains supplementary material available at 10.1186/s12951-021-00855-y.

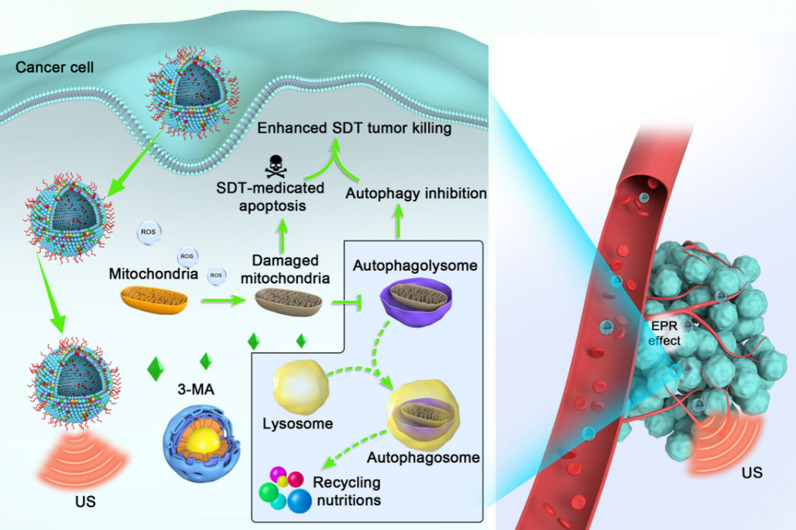

## Introduction

Autophagy is a highly conserved, lysosome-mediated degradation and recycling process by forming double-membrane autophagosomes that engulf cytoplasmic cargos for delivery to the lysosome [1, 2]. It plays a critical role in maintaining cellular homoeostasis upon various cellular stresses and diverse signals [3, 4]. Of particular importance is the role of autophagy in cancer, which is complex and paradoxical as it is responsive to suppress or support the growth of tumor cells relying on the cellular context [5–8]. Dysfunctional autophagy is closely related to the tumor progression and maintenance, and appropriate regulation of autophagic processes in cancer cells may significantly improve the sensitivity and efficacy of different therapeutics in cancer therapy [9]. Increasing evidence supports the pro-survival role of autophagy in cancer, especially stimulus-induced autophagy, by protecting tumor cells from undergoing programmed cell death [10]. One hypothesis is that intracellular metabolites derived from autophagic degradation of cellular components may be recycled to fulfill the needs of cancer-cell adaptation and growth [11]. This offers a logical basis for the inhibition of autophagy to improve sensitivity to therapeutic agents, and lays the foundation for a series of clinical trials [12–16].

In light of the encouraging clinical outcomes, care needs to be further taken to explore the role of autophagy inhibition in cancer therapy [17, 18]. Many of the steps in the autophagy pathway represent potential drug targets for negatively affecting autophagy and increasing cell death, such as the prevention of autophagosome maturation or the inhibition of autophagosome turnover [19, 20]. Multiple studies have shown that radiotherapy [21], photothermal therapy (PTT) [22, 23], and chemotherapy [24], could lead to the activation of autophagy and the combination of autophagy blockade with these therapeutics has attained a synergistic effect on cancer treatment [25]. However, all these therapeutic modalities suffer from inherent defects, including ionizing radiation, poor tissue penetration and high toxicity, which inevitably lead to reduced therapeutic effects and severe side effects [26, 27].

As an emerging and promising therapeutic approach, ultrasound (US)-triggered sonodynamic therapy (SDT) has been rapidly developed as an alternative technique for cancer treatment with specific features of non-invasiveness, deep tissue-penetrating capability, high controllability and low cost [28, 29]. US can activate sonosensitizers to generate large amounts of high energy oxygen-containing reactive oxygen species (ROS), mainly singlet oxygen (^1^O_2_) through acoustic cavitation-induced effects, such as sonoluminescence (SL) and pyrolysis, thereby inducing necrosis or apoptosis of cancer cells [30, 31]. US radiation-induced oxidative stress can cause various imbalances in cell, such as DNA damage and protein misfolding, but this intracellular macromolecular damage unfortunately may be repaired and reversed by the autophagy process, resulting in resistance to SDT-mediated apoptosis [32]. Thus, autophagy blockade provides a possibility to re-sensitize tumor and enhance the efficacy of SDT in cancer therapy.

**Fig. 1:**
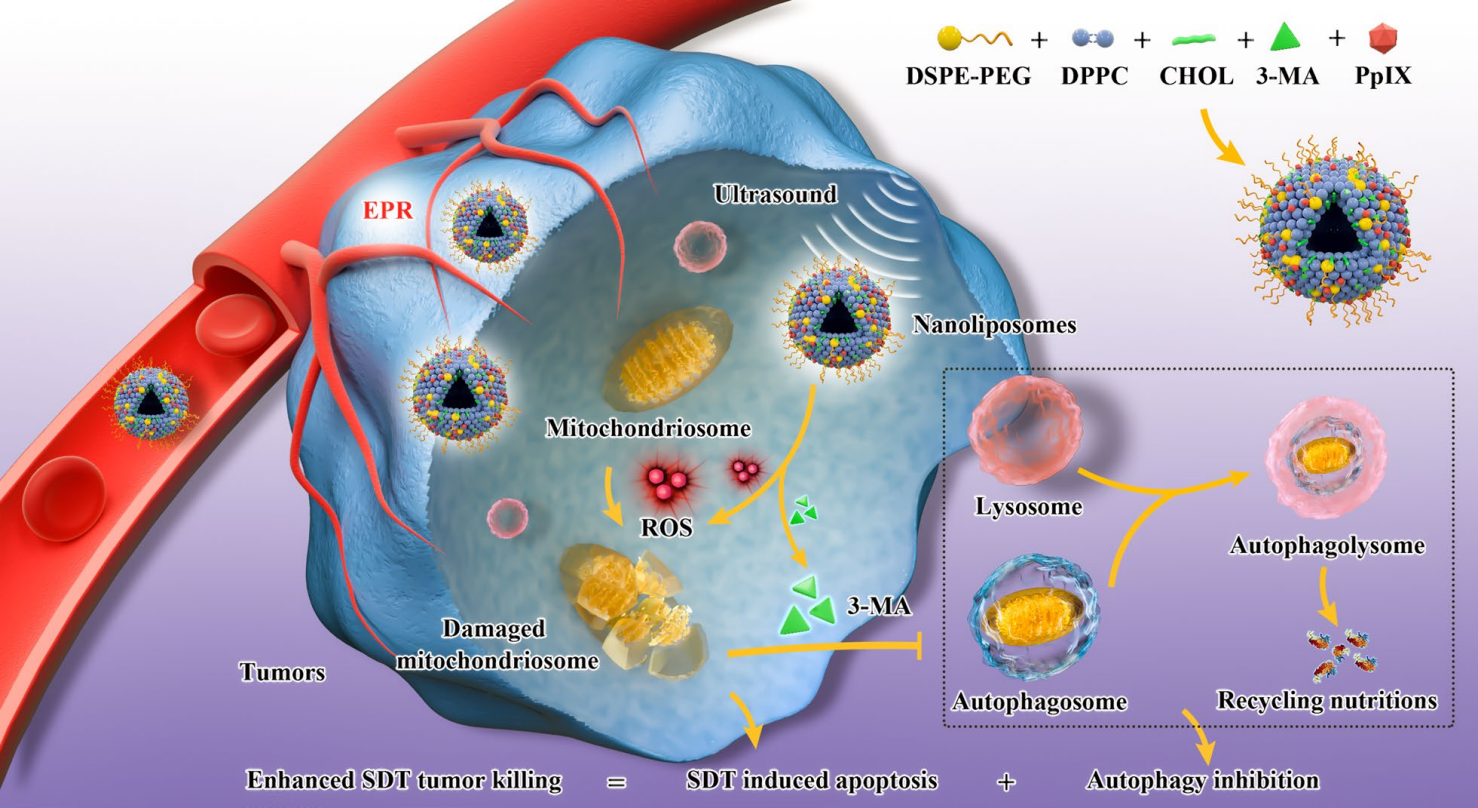
Engineering PpIX/3-MA@Lip nanosonosentizer for synergistic SDT nanotherapeutics and autophagy blockage on combating cancer. Synthetic procedure of PpIX/3-MA@Lip nanosonosensitizers and schematic illustration of “all-in-one” strategy for cellular mechanism on SDT-induced cytoprotective autophagy and autophagy inhibition-enhanced antitumor efficacy of SDT. Enhanced production of intracellular ROS radicals by PpIX sonosensitizers-based SDT induced cytoprotective pro-survival autophagy, and the integrated 3-MA inhibited the formation of autophagosomes in early-phase autophagy to eliminate the recycling nutrients for fulfilling the needs of cancer-cell adaptation and growth, which significantly induced the cancer-cell apoptosis and death

Inspired by the cross-talk between apoptosis and autophagy induced by SDT, herein we establish an “all-in-one” combined therapeutic strategy integrated with SDT and autophagy inhibition, which is successfully achieved based on the constructed autophagy inhibitor and sonosensitizers co-loaded nanoliposomes as the therapeutic nanosonosensitizers (Fig. 1). In this biosafe liposomal nanosystem, U.S. Food and Drug Administration (FDA) approved agent protoporphyrin IX (PpIX) acts as the US-responsive sonosensitizer, and 3-methyladenine (3-MA) functions as the autophagy inhibitor. SDT-induced cell damage would activate the process of autophagy to protect the cell against oxidative stress and facilitate the survival of cancer cells by attenuating apoptotic cell death, but the integrated 3-MA could inhibit the formation of autophagosomes in early-phase autophagy by regulating the phosphoinositide 3-kinase (PI3K) pathway [33]. Our results demonstrate a ROS-dependent induction of autophagy following SDT, possibly through activation of MAPK (mitogen-activated protein kinase) signaling pathway and negative regulation of AMPK (Adenosine 5 ‘-monophosphate (AMP)-activated protein kinase) signaling pathways. In particular, after combination with autophagy blockade, the synergistic antitumor effect is demonstrated to be substantially and synergistically augmented.

## Materials and methods

### Materials and reagents

Cholesterol, 1,2-distearoyl-sn-glycero-3-phosphoethanolamine-N- [amino (polyethylene glycol)2000] (DSPE-PEG-2000), Cy5.5-DSPE-PEG and dipalmitoyl phosphatidylcholine (DPPC) were obtained from Xi’an Ruixi Biological Technology Co., Ltd. Protoporphyrin IX (PpIX), 3-Methyladenine (3-MA) and N-acetylcysteine (NAC) were purchased from MedChemExpress. 2′,7′-dichlorofluorescin diacetate (DCFH-DA) was bought from Beyotime Biotechnology.2,2,6,6-Tetramethylpiperidine(TEMP),3,8-diamino-5- [3-(diethylmethylammo nio) propyl] -6-phe-nylphenanthridinium diiodide (PI), 3′,6′-di(O-acetyl) -4′,5′-bis [N, N-bis(carboxymethyl)aminomethyl] fluorescein, and tetraacetoxymethyl ester (Calcein-AM) were purchased from Dojindo Molecular Technologies. MitoScreen JC-1 Kit was bought from BD Pharmingen. Cell-counting kit-8 (CCK-8) and DAPGreen (D676) autophagy detection probe were purchased from Dojindo Molecular Technologies.

### Fabrication of PpIX/3-MA@Lip nanoliposome

PpIX/3-MA@Lip was synthesized according to the method of reverse evaporation. Firstly, PpIX and 3-MA were dissolved in methanol at a concentration of 6 mg/ml and 3 mg/ml, respectively. Then, 0.1 ml of PpIX and 0.5 ml of 3-MA were sequentially added to a chloroform solution containing DPPC, DSPE-PEG-2000, and cholesterol with a fixed weight ratio 3:1:1. After thorough mixing, reverse evaporation was conducted on a rotary evaporator at 100 mbar, 100 rpm and 60 °C for 1 h. Thereafter, the pressure was decreased to 0 mbar, and rotary evaporation was continued for 2 h at 100 rpm and 60 °C to completely remove the solvent and obtain a lipid film. 5 ml of PBS (0.01 M, pH = 7.6) was added to the lipid film, and the mixture was further rotated at 55 °C, 100 rpm for 1 h to complete the process of hydration. Finally, the liposomes were harvested by the extrusion process using a micro-extruder (200 nm and 100 nm in sequence), and further purified by dialysis. The resulting nanoliposomes were stored at 4 °C.

### Characterization

The morphology and structure of PpIX/3-MA@Lip were observed by transmission electron microscopy (TEM) scanning, which was conducted on a JEM-2100F electron microscope operated at 5 kV. UV–*vis* spectra were performed to evaluate the existence of PpIX on a UV-3101PC Shimadzu spectroscope. High-performance liquid chromatography (HPLC) was employed to determine the encapsulation efficiency of PpIX and 3-MA in the nanoliposome by comparing the area under the curve. The hydrodynamic particle size was measured by Dynamic Light Scattering (DLS) and Zeta potential was tested on a Zetasizer Nanoseries instrument (Nano ZS90).

### Detecting ^1^O_2_

ESR and DPBF. The ^1^O_2_ generation via PpIX/3-MA@Lip under US irradiation was qualitatively and quantitatively detected by electron paramagnetic resonance (ESR) and UV–*vis* spectra, respectively. In detail, PpIX/3-MA@Lip (200 μg/mL) was exposed to low-intensity focused ultrasound (LIFU, 1.0 MHz, 1.5 W cm^−2^, 50% duty cycle) for 60 s in the existence of TEMP (97 μM, Dojindo Molecular Technologies, Inc.). The generated ^1^O_2_ signal was immediately detected by the ESR spectrometer. PpIX/3-MA@Lip + TEMP group and TEMP + US group were also tested for comparison. For quantitative analysis, different concentrations of PpIX/3-MA@Lip (32 μg/ml, 16 μg/ml, and 8 μg/ml) were exposed to US irradiation (LIFU, 1.0 MHz, 1.5 W cm^−2^, 50% duty cycle) for 60 s in the existence of DPBF (Sigma-Aldrich, 40 μl, 8 mM), and the UV–*vis* spectroscope recorded the absorbance intensity at the wavelength of 410 nm. Additionally, the mixture was exposed to US irradiation every 1 min to verify its time dependence.

CLSM imaging. The detection of ^1^O_2_ production at the cellular level was performed by CLSM. After the MCF-7 cells were incubated with PpIX/3-MA@Lip for 4 h at 37 °C, the nanosonosensitizers were replaced by DCFH-DA (10 μM, Beyotime Biotechnology) and given 1 min of US irradiation (LIFU, 1.0 MHz, 1.5 W cm^−2^, 50% duty cycle). After 30 min incubation, the cells were washed three times with PBS and observed by CLSM.

### Cell culture and animals

Cell culture. Human malignant breast cell line MCF-7 was purchased from the cell bank of the Chinese Academy of Sciences. The cell line was cultured in dulbecco's modified eagle medium (DMEM) and supplemented with 10% fetal bovine serum (FBS) (Runcheng Bio-Tech Co., Ltd., Shanghai), 100 IU/mL streptomycin and 100 IU/mL penicillin at 37 °C under 5% CO_2_.

Animals. Female Kunming mice and female BALB/c nude mice aged 6–8 weeks were obtained from Shanghai Experimental Animal Center (Shanghai, China). All animal experiments were performed according to experimental animal’s administrative regulation of Hubei Province and guidelines for experimental animal’s ethical committee of Huazhong University of Science and Technology.

### Autophagy analysis

CLSM imaging. To stain autophagosomes and autolysosomes, MCF-7 cancer cells were incubated with DAPGreen (5 nM) in the culture medium for 30 min at 37 °C followed by two washes with medium. The cells were observed by CLSM at various time points after SDT and other different treatments.

Flow cytometric analysis. DAPGreen was also used to stain the autophagosomes and autolysosomes of MCF-7 cancer cells for quantitative flow cytometry analysis. At 0.5 h, 2 h and 4 h after SDT treatment, the cancer cells were rinsed with medium twice and detected using a flow cytometer.

Western blot assay. After collecting the cells, the protein was extracted with RIPA buffer containing a phosphatase inhibitor and a protease inhibitor (Beyotime Biotechnology, Shanghai, China), and then the protein concentration was determined by a bicinchoninic assay (BCA) (Pierce Biotechnology, Inc., Rockford, IL). Thereafter, the proteins were separated on 12% SDS-PAGE and transferred onto a onto a polyvinylidene difluoride (PVDF) membrane (Millipore, Bedford, USA). Membranes were blocked with Tris-buffered saline containing 0.1% Tween-20 and 5% nonfat dry milk, and incubated with diluted primary antibody (anti-LC3) (Cell Signaling Technology, Boston, USA) overnight at 4 °C. After extensive washing, membranes continued to incubate with anti-rabbit secondary antibody labeled fluorescence (Wuhan servicebio technology Co., Ltd) for 2 h at room temperature, and finally visualized with enhanced chemoluminescence kit via ImageQuant LAS 4000 system (GE Healthcare Life Sciences, USA).

TEM observation. The collected cells were fixed with 2.5% glutaraldehyde, then dehydrated with increased concentrations of ethanol and embedded in an epoxy resin. The resin containing the sample was cut into a 70-nm thick films. After staining with 4% uranyl acetate and 2.5% glutaraldehyde, the films were imaged on the JEM-1410 TEM at 100 kV.

### RNA-sequencing assay and bioinformatics analysis

RNA high throughput sequencing was performed by Cloud-Seq Biotech (Shanghai, China). Total RNAs from the MCF-7 cells with or without SDT treatment were extracted with TRIzol (Invitrogen). After constructing and evaluating the RNA libraries by using NEBNext® Ultra™ II Directional RNA Library Prep Kit (New England Biolabs, Inc., Massachusetts, USA) and BioAnalyzer 2100 system (Agilent Technologies, Inc., USA), library sequencing was conducted on an illumina Hiseq 4000 machines with 150 bp paired end reads. The gene level FPKM values were obtained using cuffdiff software based on the Ensembl gtf gene annotation file. As the expression profiles of mRNA, FPKM values were used to calculate fold change and p-value of differentially expressed mRNAs. GO terms that annotate an enriched gene list with significant *P* values < 0.05 were identified using GO-Term Finder. KEGG pathway enrichment analysis of differentially expressed mRNAs was performed to determine the pathways in which they were involved using DAVID (the Database for Annotation, Visualization and Integrated Discovery; https://david.ncif crf.gov/). *P* values < 0.05 as the threshold for significant enrichment.

### In vitro cytotoxicity assay

CLSM imaging. For intuitively observing the therapeutic effect on CLSM, MCF-7 cells were seeded in confocal dishes. After US irradiation, the viable and dead cells were stained by calcein-AM (10 μM) and PI (15 μM) for 15 min in the dark. The staining solution was then removed, and the cells were rinsed with cold PBS twice gently before confocal microscopy experiments.

Flow cytometric analysis. Apoptosis and the destruction of mitochondrial membrane potential (MMP) were studied using flow cytometry with the Annexin V- FITC/PI and JC-1staining assay kit according to the manufacturer’s instructions, respectively. Briefly, cells (1 × 10^6^) were resuspended in 200 μl of binding buffer containing dyes and incubated for 15 min at room temperature in the dark, followed by flow cytometric analysis. Regional statistical analysis was conducted by using Flow Jo software.

CCK-8 assay. The effect of autophagy modulators combined with SDT on the cell viability of MCF-7 cells was evaluated by the typical CCK-8 assay. MCF-7 cells were seeded into 96-well plates at a density of 2 × 10^4^ cells per well and divided into different treatment groups, including control, PpIX@Lip, PpIX/3-MA@Lip, US only, PpIX@Lip + US, and PpIX/3-MA@Lip + US. In addition, MCF-7 cells were treated with different concentrations of PpIX/3-MA@Lip (10, 20, 40, 80, and 160 μg/ml) with or without US irradiation. After 24 h of treatment, the culture mediums were discarded and cell viabilities were evaluated using CCK-8. The optical density was read at 450 nm using an automated microplate reader (Bio-TekELx800, USA).

### In vivo biosafety assay

For in vivo biosafety evaluation, 25 healthy female Kunming mice were randomly divided into five groups (n = 5 in each group), including control, PpIX/3-MA@Lip, US only, PpIX@Lip + US, and PpIX/3-MA@Lip + US. Different kinds of nanoliposomes were intravenously injected into the mice (the PpIX dose of 10 mg/kg and 3-MA dose of 8 mg/kg, 150 μl) and US irradiation (LIFU, 1.0 MHz, 2.5 W cm^−2^, 50% duty cycle, 5 min) was performed. The control group was not given any treatment. After four weeks of observation, the mice finally were sacrificed to acquire blood samples and main organs (heart, liver, spleen, lung, and kidney) for biosafety evaluation.

### In vivo autophagy inhibition-sensitized SDT antitumor performance

All MCF-7 tumor-bearing female BALB/c nude mice models applied for synergistic treatment were established by subcutaneous tumor xenotransplantation. 6 weeks old mouse models bearing MCF-7 tumors were randomly divided into five groups (n = 5 in each group), including PBS, PpIX/3-MA@Lip, US only, PpIX@Lip + US, and PpIX/3-MA@Lip + US groups. Administration of tail-vein infusion (the PpIX dose of 10 mg/kg and 3-MA dose of 8 mg/kg, 150 μl) and US irradiation (LIFU, 1.0 MHz, 2.5 W cm^−2^, 50% duty cycle, 5 min) were performed when tumors reached ~ 70 mm^3^. The injections were performed twice on day 0 and day 2, and US irradiation were conducted three times on the first day, the third day and the fifth day. The body weight and tumor volume were monitored every two days for half a month. Tumor volume was measured by caliper measurements using the formula (width^2^ × length) / 2. Tumor-inhibition rate was defined as (1 − V/V0) × 100% (V0: tumor volume of control group). After the 15 days’ therapeutic period, the tumor was dissected and further stained by H&E, TUNEL, and LC3 for systematical pathological analysis.

### Statistical analysis

Statistical comparisons were conducted by Student’s t test with a setting significance of *P < 0.05, **P < 0.01, ***P < 0.001. All quantitative data were expressed as mean ± standard deviation (s.d.) of at least three independent measurements. All statistical analyses were performed by using SPSS version 20.0 software (SPSS, Chicago, IL).

## Results and discussion

**Fig. 2:**
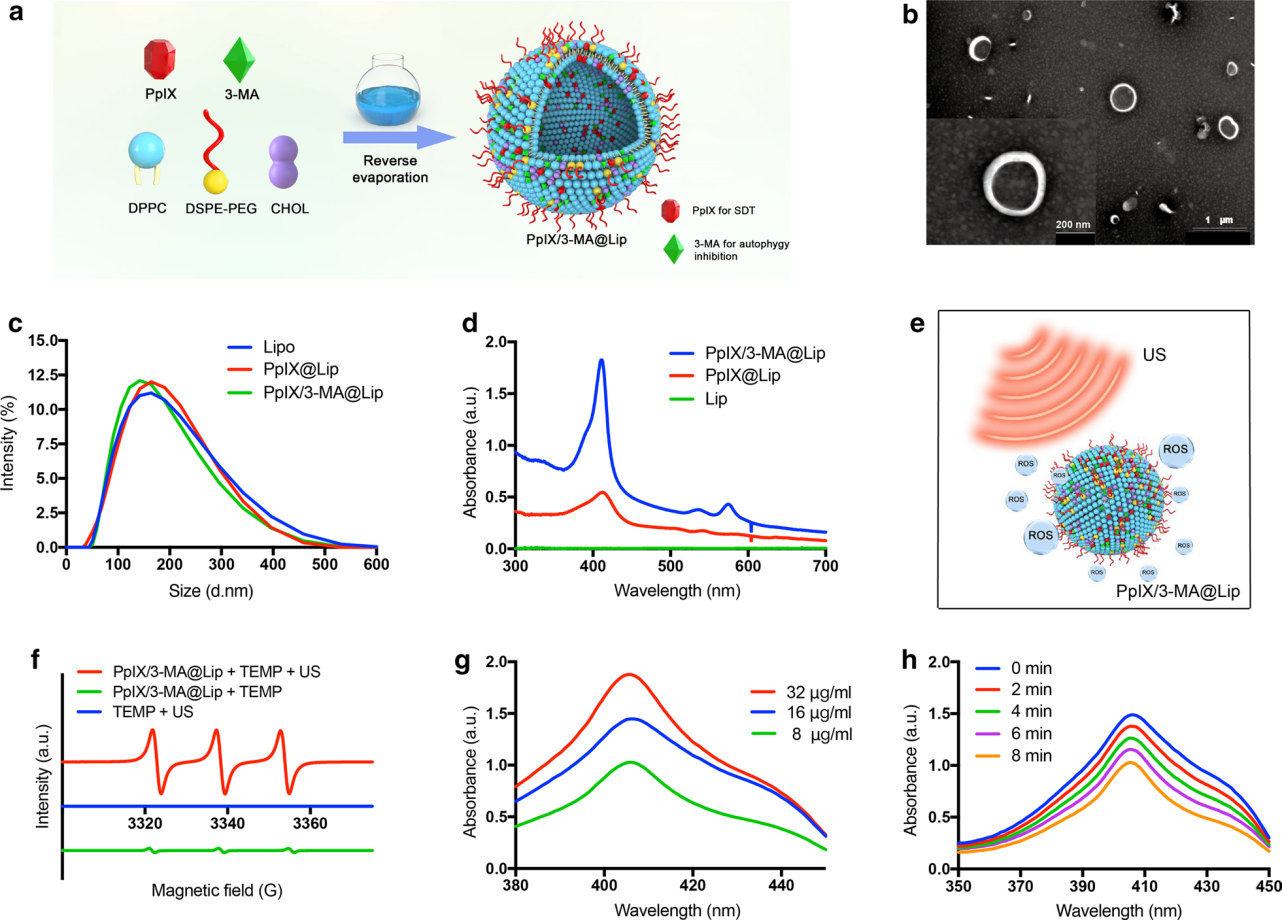
Structure, composition and physiochemical property characterizations of PpIX/3-MA@Lip nanoliposomes. **a** Synthetic procedure of PpIX/3-MA@Lip nanosonosensitizers. **b** TEM image of PpIX/3-MA@Lip nanoliposomes. Scale bar = 200 nm. **c** Hydrodynamic diameters, and **d** UV–*vis* spectra of Lip, PpIX@Lip and PpIX/3-MA@Lip. **e** Schematic illustration of US-triggered ROS production based on PpIX/3-MA@Lip. **f** ESR spectra of PpIX/3-MA@Lip with or without US irradiation (1.0 MHz, 1.5 W cm^−2^, 1 min). TEMP with US irradiation was used for control comparison. **g** Concentration and **h** time dependent DPBF absorption spectra of PpIX/3-MA@Lip nanoliposome under US irradiation (1.0 MHz, 1.5 W cm^−2^, 1 min)

Nanoliposomes co-loaded with two hydrophobic agents were synthesized by a typical reverse evaporation method [34], which encapsulated sonosensitizers (PpIX) and autophagy inhibitor (3-MA), designated as PpIX/3-MA@Lip (Fig. 2a). TEM images revealed that the obtained PpIX/3-MA@Lip nanoliposomes had well dispersion in aqueous solution and appeared as quasi-spheres with similar size (Fig. 2b). Dynamic light scattering (DLS) measurement was performed to determine the size and Zeta potential of these nanoliposomes, confirming that they had an average hydrodynamic diameter of 143.2 nm (Fig. 2c) and an average surface charge of − 35 mV (Additional file 1: Figure S1). The drug-loaded nanoliposomes could maintain favorable structural stability under physiological conditions, as shown by the negligible size and Zeta potential change in phosphate buffered saline (PBS) at 4 °C for 7 days (Additional file 1: Figure S2). Compared to pure liposome, PpIX/3-MA@Lip and PpIX@Lip nanoliposomes exhibited strong absorption peaks at 410 nm in UV–*vis*-NIR absorption spectrum, indicating the successful encapsulation of PpIX into nanoparticles (Fig. 2d), which was also validated by the results of high-performance liquid chromatography (HPLC) (Additional file 1: Figure S3a, b). The efficient co-loading of 3-MA was further confirmed by HPLC (Additional file 1: Figure S3c, d). The encapsulation efficiency and loading capacity of PpIX and 3-MA in nanoliposomes varied with different dose concentrations (Additional file 1: Figure S3e, f). It has been demonstrated that SDT can activate nanosonosensitizers to generate large amounts of high energy oxygen-containing ROS-based radicals (^1^O_2_) for inducing necrosis or apoptosis of cancer cells under US irradiation (Fig. 2e) [35]. In order to investigate the capability of PpIX/3-MA@Lip to produce ROS, we adopted electron spin resonance (ESR) and a 1,3-diphenyliso-benzofuran (DPBF) assay to qualitatively and quantitatively monitor the ROS generation, respectively [36, 37]. For ^1^O_2_ detection, 2,2,6,6-tetramethylpiperide (TEMP) as the trapping agent was mixed with PpIX/3-MA@Lip and irradiated by low-intensity focused ultrasound (LIFU, 1.0 MHz, 1.5 W cm^−2^, 1 min). As shown in Fig. 2f, PpIX/3-MA@Lip had a much higher ^1^O_2_ generation efficiency than TEMP under the irradiation of US. In addition, the generation of ^1^O_2_ can oxidatively degrade DPBF, resulting in decreased absorbance intensity at the wavelength of 410 nm in UV–*vis* spectrum, which can be applied for quantitative analysis of ^1^O_2_. It can be found that the absorbance intensity of DPBF exhibited concentration dependence of nanosonosensitizers (Fig. 2g), and as the US irradiation duration prolonged, the absorbance intensity of DPBF gradually decreased in the presence of PpIX/3-MA@Lip (Fig. 2h; Additional file 1: Figure S4), demonstrating the efficient production of ^1^O_2_ during SDT process.

**Fig. 3:**
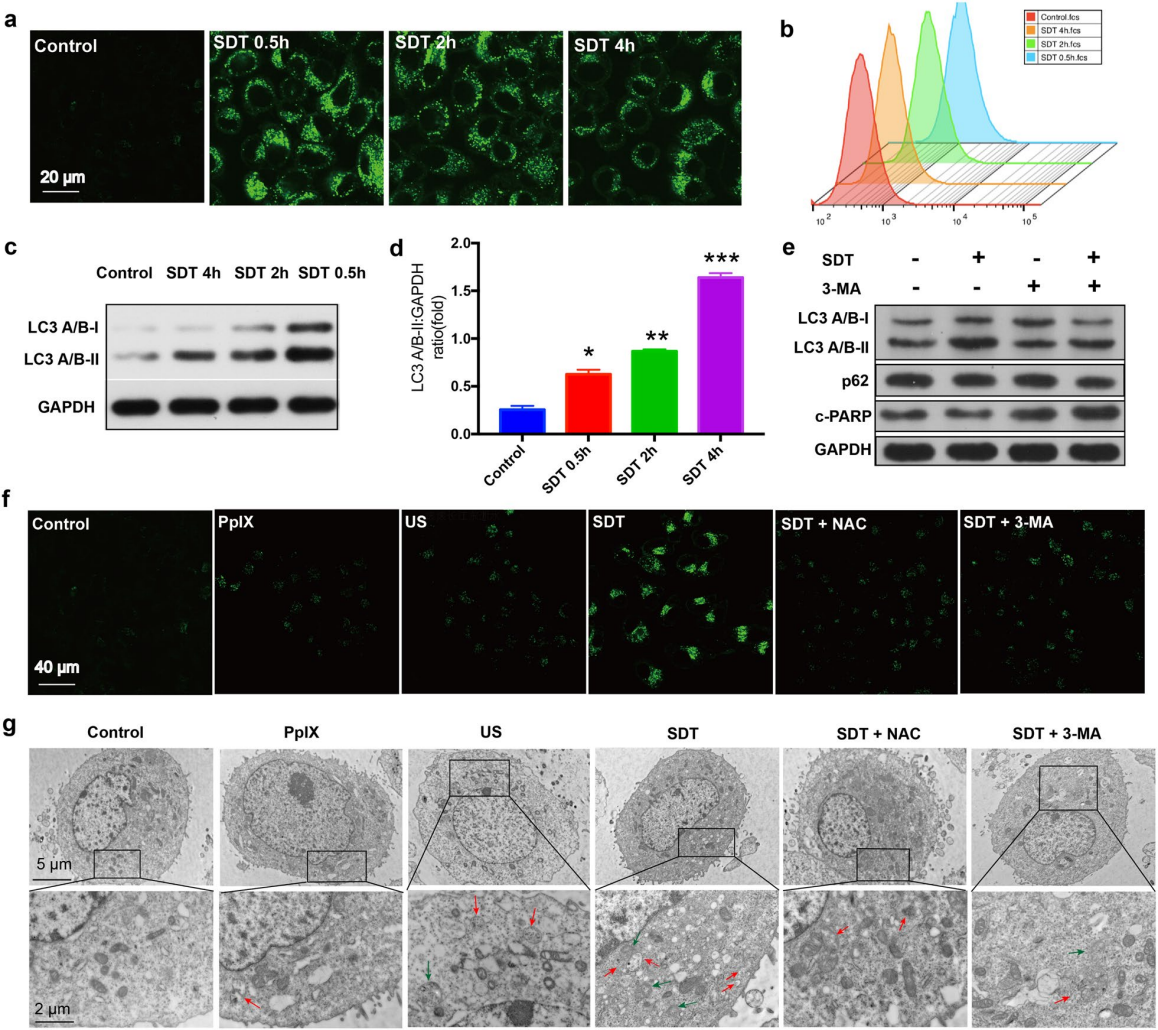
In vitro evaluation of autophagy induction by SDT and autophagy inhibition by 3-MA. **a**, **b** CLSM images and flow cytometry analysis of MCF-7 breast cancer cells stained by DAPGreen for monitoring cytoprotective autophagy induced by SDT treatment at different time points. **c**, **d** Western blot analysis for LC3-I and LC3-II and corresponding quantitative data of LC3-II relative to GAPGH. GAPDH expression level serves as the loading control. Quantitative data are presented as means ± s.d. (n = 3) *P < 0.05, **P < 0.01, and *** *P* < 0.001*.* **e** Western blot analysis for the expressions of autophagy indicator proteins LC3B and p62, and apoptosis indicator protein c-PARP in MCF-7 cells after different treatments. GAPDH was used as a loading control. **f** CLSM images of MCF-7 cells stained by DAPGreen treated with nothing (control), PpIX, US, SDT, SDT + NAC, and SDT + 3-MA. **g** TEM images of MCF-7 cells treated with nothing (control), PpIX, US, SDT, SDT + NAC, and SDT + 3-MA. Red arrows indicate autophagosome and green arrows indicate autolysosome

Most anticancer agents inevitably cause cellular stress, and autophagy is usually activated in cancer cells after varied treatments. As an exogenous stimulus, SDT can produce a large amount of ROS and cause certain imbalances in cancer cells, which may induce the occurrence of cytoprotective autophagy. To investigate the effect of SDT on autophagy and autophagic flux in MCF-7 cancer cells, we initially used fluorescent dyes DAPGreen to detect the formation of autophagosome and autolysosome by confocal laser scanning microscopy (CLSM) and flow cytometry analysis. DAPGreen is favorable for monitoring early and late-phase autophagy by entering the bilayer membrane structure when autophagosomes are formed, which emits strong fluorescence in a hydrophobic environment [38, 39]. Compared with control, SDT resulted in stronger fluorescence brightness at 0.5 h post SDT treatment, demonstrating that the quantities of autophagosomes are increased after SDT treatment. After that, the fluorescence brightness gradually weakened (Fig. 3a), probably due to the degradation of autophagosomes by lysosomes. This phenomenon was also confirmed by flow cytometry analysis for the determination of fluorescence-intensity changes (Fig. 3b), indicating that SDT caused autophagy activation and accelerated autophagosome accumulation in MCF-7 cancer cells. However, both increased autophagosome formation and decreased autophagosome turnover may result in enhanced autophagosome accumulation, which should be determined to be involved in SDT process. To further provide additional convincing evidence of SDT-induced autophagy, the cellular level of autophagy marker protein microtubule-associated protein 1 light chain 3 (LC3) relative to GAPDH was determined via western blot analysis [40]. As a typical autophagy-related protein, LC3-II undergoes post-translational modifications resulting from the formation of autophagosomes and commonly serves as an autophagysome marker [41]. In consistence with DAPGreen-staining results, compared with control, SDT induced more significantly increased LC3-II protein expression at 0.5 h post SDT treatment. Moreover, the level of increased LC3-II protein which conversed from LC3-I was gradually reduced from 0.5 h to 4 h post SDT treatment (Fig. 3c, d). These results suggested that SDT promoted autophagosome formation rather than prevented degradation of autophagosomal substrates downstream, meaning normal lysosomal function after SDT. To further confirm this mechanism, we also measured the level of sequestosome 1(SQSTM1/p62), a specific protein substrate that can be selectively intergrated into the forming autophagosome and degraded in autolysosomes [42]. Western blot assay exhibited that the expression of p62 was significantly downregulated in SDT group (Fig. 3e; Additional file 1: Figure S5), further evidencing that the elevation of oxidative stress by SDT could induce cytoprotective autophagy. Taken together, these results solidly demonstrated that the SDT-induced autophagy was achieved by increasing autophagosome formation rather than autophagosome turnover. Furthermore, TEM was used for direct observation of the formed autophagosomes and autolysosomes [14]. As shown in Fig. 3g, without US irradiation, there were no autophagic vacuoles that could be observed in the control group. Both the US group and the PpIX group induced a weak autophagy. Comparatively, multiple bilayer membranes autophagosomes and monolayers autolysosomes were clearly revealed after SDT treatment. However, highly weak fluorescence intensity in CLSM images (Fig. 3f) and much less autophagic vacuoles in TEM images (Fig. 3g) were observed when SDT triggered intracellular ROS were eliminated by ROS scavenger N-acetylcysteine (NAC), indicating the intracellular ROS elevation was involved in SDT-induced cell autophagy. Altogether, these results strongly presage that the SDT effect of nanosonosensitizers activates the process of autophagy and enhances autophagosomes accumulation in MCF-7 cancer cells.

To further determine whether autophagy inhibitor prevented autophagy induced by SDT, we treated MCF-7 cancer cells with PpIX/3-MA@lip under US irradiation. The result of western blot analysis demonstrated that 3-MA efficiently prevented the conversion of LC3-I to LC3-II protein in 3-MA and synergistic therapy groups, revealing the quantity reduction of autophagosomes in cancer cells (Fig. 3e; Additional file 1: Figure S5). The abundance of p62 was also decreased in 3-MA and synergistic therapy groups, demonstrating that 3-MA could inhibit autophagy by preventing the formation of autophagic machinery and substrates (Fig. 3e; Additional file 1: Figure S5). Moreover, 3-MA-incubated MCF-7 cells presented much less green fluorescent punctate dots compared with SDT group in CLSM images (Fig. 3f). In addition, TEM observation also indicated that 3-MA blocked the formation of autophagosomes (Fig. 3g). The significant decrease of autophagic vesicles in SDT/3-MA treated cells is attributed to 3-MA for arresting the formation of autophagosomes in the early stages of the autophagy process via its inhibitory effect on class III PI3K. All the results provide reliable evidence to support the differentiation between activation and inhibition of autophagy. SDT boosts the whole autophagic flux and 3-MA blocks autophagosome formation. The synergy between SDT and 3-MA effectively prevents the cancer cells from eliminating damaged proteins and organelles to achieve intracellular organelle homeostasis, which contributes to augment oxidative stress damage induced by SDT. Given the above evidenced findings and the critical role of autophagy in tumor progression and maintenance, the inhibition of autophagy combined with conventional SDT is highly advantageous for tumor treatments.

**Fig. 4:**
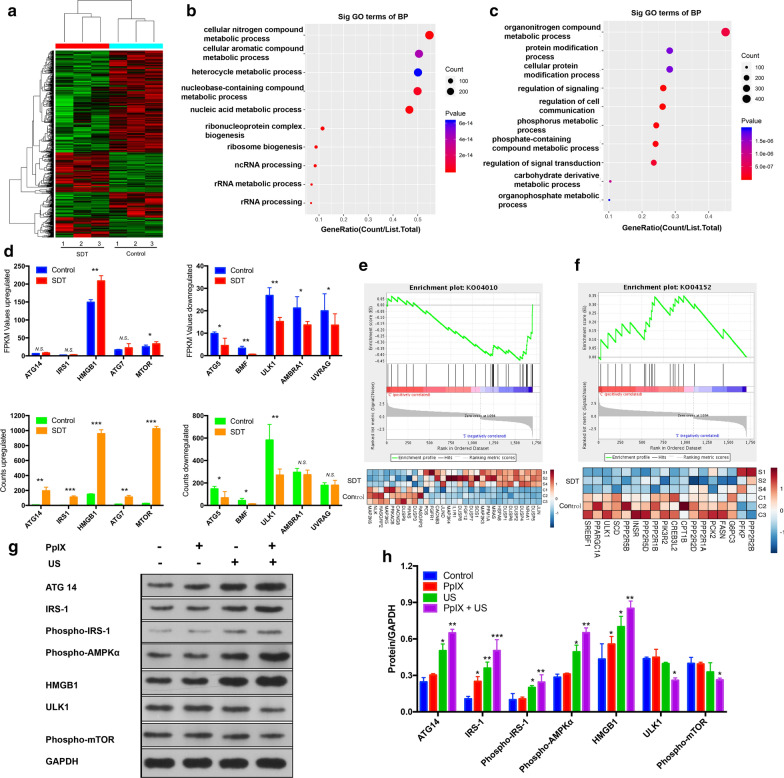
Transcriptome high throughput sequencing of mRNA expressions with SDT. **a** Heat map diagram of 1095 differentially expressing mRNAs including 561 up-regulations and 534 down-regulations. **b**, **c** Corresponding gene expression ratios for up-regulated and down-regulated GO classifications. **d** FPKM value and counts of typical mRNA expressions associated with cytoprotective autophagy treated with SDT. Values are presented as means ± s.d. (n = 3) *P < 0.05, **P < 0.01, and *n.s.* for non-significant. **e**, **f** Gene set enrichment analysis (GSEA) for autophagy-related signaling pathway after SDT. **g**, **h** Western blot verification analysis for the expressions of autophagy-related proteins after SDT. GAPDH was used as a loading control. Quantitative data are presented as means ± s.d. (n = 3) *P < 0.05, **P < 0.01, and *** *P* < 0.001

High-throughput sequencing was performed to explore the mRNA variations of MCF-7 cancer cells with or without SDT treatment [43, 44]. A total of 1095 mRNAs were critically involved in possible biological effects in the process of SDT. With a strict cutoff value of twofold, 561 and 534 mRNAs were found to have been significantly and differentially upregulated and downregulated (*P* < 0.05) respectively by comparing the SDT samples to the untreated control group, respectively (Fig. 4a; Additional file 1: Figure S6). To better understand the biological functions of mRNAs, Gene Ontology (GO) analysis (biological process, cellular component and molecular function) was conducted on the mRNAs with significantly altered expression level (Fig. 4b, c; Additional file 1: Figure S7). Corresponding gene expression ratios were also depicted to demonstrate the co-relationship among the biological processes from which an overall percentage of 20% could be acquired (Fig. 4b, c). From all expressed mRNAs, relevant information associated with the process of autophagy was extracted and compared according to their fragments per kilobase of transcript per million mapped fragments value (FPKM value) and count value (Fig. 4d). Kyoto Encyclopedia of Genes and Genomes (KEGG) signaling pathway analysis indicated that the differentially expressed mRNAs were probably associated with autophagy-related signaling pathway after SDT, including MAPK signaling pathway and AMPK signaling pathway (Additional file 1: Figure S8). Additionally, gene set enrichment analysis (GSEA) [45] demonstrated that the increased expression of mRNAs was significantly enriched in signaling pathways that promoted autophagy, and decreased expression of mRNAs was significantly enriched in signaling pathways that inhibited autophagy (Fig. 4e, f).

The formation and turnover of the autophagosome are closely related to evolutionarily conserved autophagy-related genes (ATGs) [46]. The entire autophagy process is divided into five distinguishable stages: initiation, autophagosome nucleation, autophagosome elongation, autophagosome and lysosome fusion, and degradation of intravesicular cargos. Insulin or insulin-like growth factor regulates mTOR through PI3K(I). The autophosphorylation of insulin receptor located in tyrosine can amplify and phosphorylate insulin receptor substrate 1 (IRS1), then inhibit the downstream PKB/Akt signal, thus inhibit the expression and phosphorylation of mTOR, and regulate autophagy with positive feedback. The release of the inhibitory effect of mTOR on the ULK1 complex (comprising ULK1, ULK2, FIP200, ATG101 and ATG13) enables the autophagy process to be initiated. The ULK1 complex induces autophagosome nucleation, which is then mediated by a class III PI3K complex consisting of VPS34, ATG14, UVRAG, AMBRA1. The ATG5-ATG12 complex binds to ATG16 to expand the autophagosome membrane, and ATG4B combines with ATG7 to conjugate LC3I and phosphatidylethanolamine (PE) to form LC3-II. Through transcriptome analysis of SDT mRNA expression sequencing, it has been found that SDT is intently related to the initiation of autophagy and nucleation of autophagosomes, and SDT-induced partial genetic changes in the autophagy pathway potentially represent druggable targets and provide ways to negatively influence autophagy.

In addition to the results of high-throughput mRNA sequencing, another important work is that a much better understanding of how SDT regulates changes in the expression of autophagy-related protein through western blot analysis. Consistent with increased rather than decreased autophagy, it has been found that SDT enhanced intracellular ROS production, increased the level of ATG 14 and IRS-1, activated the phosphorylation of IRS-1 and AMPK, decreased the level of ULK1, and downregulated the mTOR pathway (Fig. 4g, h). Furthermore, as a key molecule to promote autophagy and inhibit apoptosis by binding to Beclin-1, the expression and cytosolic levels of high mobility group box 1 (HMGB1) also increased in response to SDT stress (Fig. 4g, h). These results manifest the possibility that SDT leads to a remarkable upregulation of autophagy, supporting its role as a key survival mechanism in breast cancer.

**Fig. 5:**
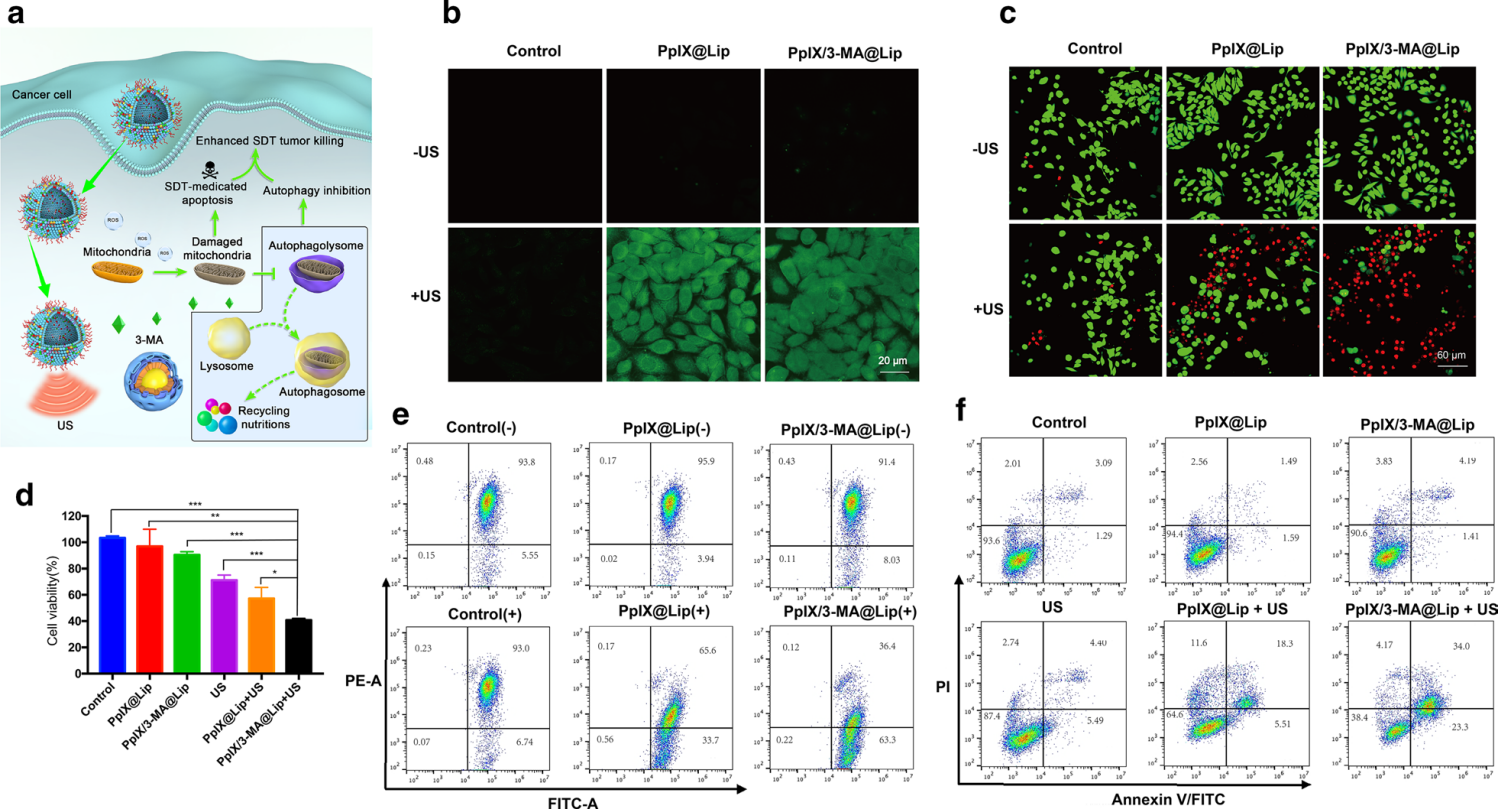
In vitro autophagy inhibition-enhanced SDT efficacy by accelerating cell apoptosis. **a** The synergistic anti-tumor mechanism of SDT and autophagy inhibition. **b**, **c** CLSM images of MCF-7 cells stained with DCFH-DA for ROS detection, and Calcein-AM/PI for live /dead cells identification after different treatments. **d** Viability assays of cancer cells. Values are presented as means ± s.d. (n = 3) *P < 0.05, **P < 0.01, ***P < 0.001. **e**, **f** Flow cytometry analysis for the destruction of MMP and evaluation of apoptosis at various stages after different treatments

After verification of SDT for upregulating cellular autophagic level, we moved on to reveal if the inhibition of autophagy could contribute to their cell-killing efficacy as hypothesized (Fig. 5a). CLSM revealed that most nanoliposomes could be rapidly internalized by MCF-7 cancer cells within 4 h co-incubation (Additional file 1: Figure S9), and the effective intracellular uptake of nanosonosensitizers guaranteed efficient SDT treatment under US radiation. In order to explore the underlying mechanism of killing cancer cell by US combined with PpIX/3-MA@lip nanosonosensitizer at the cellular level, a ROS fluorescence probe 2′,7′-dichlorofluorescin diacetate (DCFH-DA) was applied to illustrate the ROS production [35]. As indicated in Fig. 5b, the green fluorescence was hardly observed in the control, PpIX@lip-, or PpIX/3-MA@lip-treated cancer cells. Contrarily, it was found that PpIX@lip and PpIX/3-MA@lip combined with US irradiation exhibited strong green fluorescence due to the massive intracellular ROS production by the SDT effect. The quantitative analysis of ROS-generating capability of nanoliposomes was also performed (Additional file 1: Figure S10).

Combined with ESR result (Fig. 2f), it was assumed that PpIX/3-MA@lip as sonosensitizers could generate ROS (^1^O_2_) under US irradiation to induce cytotoxic effect and further achieve the SDT therapeutic function. To exam whether PpIX/3-MA@lip could be safely and effectively applied for treating cancer under US irradiation, we evaluated the cytotoxicity via the typical cell-counting kit-8 (CCK-8) assay. As shown in Fig. 5d, the sole PpIX@lip, PpIX/3-MA@lip, or US treatment did not induce significant cytotoxicity. Comparatively, US irradiation (1.0 MHz, 1.5 W cm^−2^, 1 min, 50% duty cycle) in combination with PpIX @lip and PpIX/3-MA@lip induced massive cell death, and PpIX/3-MA@lip achieved higher therapeutic efficacy, indicating that the autophagy inhibition markedly enhanced SDT efficacy by synergistically enhancing cell apoptosis. In addition, the cell viability of MCF-7 gradually decreased in a dose-dependent pattern (Additional file 1: Figure S11). The PpIX/3-MA@lip present decreased cell viabilities at ascending concentrations under US irradiation, while much higher cell viabilities were observed at corresponding concentrations without US irradiation (Additional file 1: Figure S11). These results were further validated by CLSM fluorescence images of MCF-7 cells co-staining with calcein acetoxymethyl ester (calcein-AM) and propidium iodide (PI) solution to visually differentiate between live (green color) and dead (red color) cells, which further proved the excellent synergistically enhanced SDT efficacy of PpIX/3-MA@lip under US irradiation (Fig. 5c).

The underlying molecular mechanism by which autophagy inhibition participats in SDT-induced cell death was further investigated by western blot analysis (Fig. 3e; Additional file 1: Figure S5). Cleaved Caspase 3 and 7 (c-CAS3/7), the major terminal cleavage enzyme during apoptosis, are typical pro-apoptotic markers [47]. 3-MA, which blocks autophagic formation by regulating the PI3K pathway, further enhanced caspase accumulation in the cells treated with SDT, as compared to the cells treated with 3-MA or SDT only, confirming the apoptotic pathway of breast cancer cells after synergistic therapy. As a cleavage substrate for caspase, cleaved poly-ADP-ribose polymerase (c-PARP) is extensively involved in cellular responses to DNA damage and DNA metabolism [48]. Compared with other treatment groups, the combinational treatment significantly upregulated c-PARP, demonstrating that the synergistic effect between SDT and autophagy block may cause obvious DNA strand breaks and subsequent epigenetic regulation of abnormal chromatin structure, thereby guiding cancer cells into the apoptotic pathway.

The cell apoptosis level was further checked by flow cytometry analysis. Mitochondrial as the primary targets in the process of SDT, is often accompanied by the destruction of mitochondrial membrane potential (MMP) in the process of apoptosis, which is broadly regarded as one of the earliest events in the process of apoptosis [49]. JC-1 is a lipophilic fluorochrome that is exploited to investigate the changes in MMP [50]. After stained with JC-1, MCF-7 cancer cells incubated with PpIX/3-MA@lip presented a lower red fluorescence signal intensity than PpIX@lip under US irradiation (Fig. 5e), suggesting the enhanced enormous loss of MMP and mitochondrial damage. In addition, apoptosis evaluation at different stages was also performed by Annexin V-FITC and Propidium Iodide (PI) staining (Fig. 5f). SDT assisted with PpIX/3-MA@lip induced high levels of early and late apoptosis (57.3%), which was much higher than PpIX@Lip (23.8%). Taken together, these data provided solid evidences to demonstrate that the autophagy inhibition could contribute to SDT-induced cytotoxicity through enhancing cell apoptosis.

**Fig. 6:**
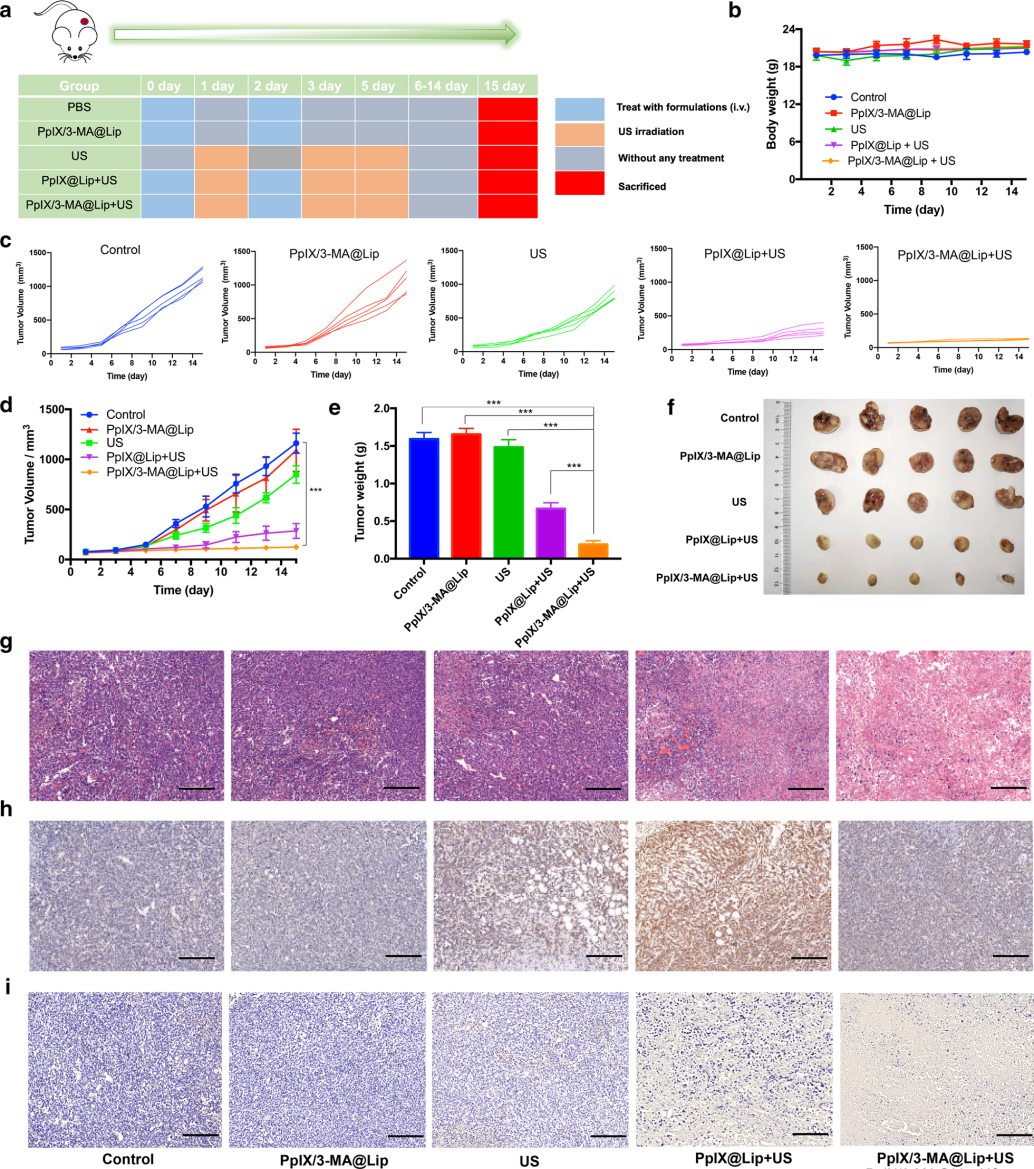
In vivo synergistic antitumor efficiency of SDT and autophagy inhibition. **a** Schematic illustration of tumor-bearing mice treated with different formulations under US irradiation (1.0 MHz, 2.5 W cm^−2^, 50% duty cycle, 5 min) (n = 5). **b** Curves of body weight of tumor-bearing mice with different treatments during 15 days. **c**, **d** Tumor-volume evolutions during the therapeutic period. **e** Average tumor weights and **f** photograph images of excised tumors from different groups on the 15th day. Values are presented as means ± s.d. (n = 5) ***P < 0.001. **g** H&E, h) LC3, and **i** TUNEL staining images of excised tumors from different groups. Scale bar = 100 μm

Encouraged by the intriguing in vitro enhanced SDT efficacy, we further assessed the in vivo antitumor efficacy of autophagy blockage combined with SDT treatment. BALB/c nude mice were implanted with MCF-7 tumors before intravenous administration of nanoliposome (Fig. 6a). Before conductig the therapeutic experiment, the accumulation of Cy5.5-labelled PpIX/3-MA@Lip in tumor tissue and majour organs was initially explored by utilizing the in vivo fluorescence imaging system (IVFIS). It revealed that PpIX/3-MA@Lip nanosonosensitisers located into breast tumor foci after i.v. administration via the typical enhanced penetration and retention (EPR) effect (Additional file 1: Figure S12), which was mainly attributed to high stability and long blood-circulation duration of nanosonosensitisers. When the tumor volume reached nearly 70 mm^3^, MCF-7-tumor-bearing mice were randomly divided into five groups (n = 5 per group), including PBS, PpIX/3-MA@Lip, US only, PpIX@Lip + US, and PpIX/3-MA@Lip + US groups. After the intravenous injection of nanoliposome for 24 h, the tumor was irradiated with US (1.0 MHz, 2.5 W cm^−2^, 50% duty cycle, 5 min) and repeated on the third and fifth day (Fig. 6a). During 15 days of the therapeutic period, the body weight of mice among all groups showed no significant changes, suggesting that no severe side effects were induced by the combined therapy (Fig. 6b). Compared with the control group, the tumor growth was suppressed to some extent in the PpIX/3-MA@Lip group, US group, and PpIX@Lip + US group, while the PpIX/3-MA@Lip + US group achieved the most potent efficiency in tumor suppression (Fig. 6c–e). The tumor-inhibition rate of PpIX/3-MA@Lip + US group reached 89.32%, substantially higher than those of PpIX@Lip + US group (75.46%), US only group (26.98%), and PpIX/3-MA@Lip group (6.13%). Photographic images of the dissected tumor at the end of treatments also visually demonstrated the smallest tumor size in the PpIX/3-MA@Lip + US group compared to the other four groups (Fig. 6f).

The mechanism of enhanced antitumor efficiency by combing SDT and autophagy blockade was further revealed by pathological sections stained by haematoxylin–eosin (H&E), LC3, and TdT-mediated dUTP nickend labelling (TUNEL). In H&E-stained images, the tumor was destroyed slightly in PpIX/3-MA@Lip, US or PpIX@Lip + US-treated mice. Conversely, PpIX/3-MA@Lip + US group exhibited the most severe tumor necrosis (Fig. 6g). Remarkably, a wide distribution of endogenous LC3 was visualized in PpIX@Lip + US group, revealing the induction of autophagy by SDT. Comparatively, the PpIX/3-MA@Lip + US group showed less LC3 distribution due to autophagy inhibition by 3-MA (Fig. 6h). From the TUNEL assay result, the number of apoptotic cells as stained dark-brown in the PpIX/3-MA@Lip + US group was much more than those of the other control groups (Fig. 6i). These results were consistent with in vitro cell experiments, which were designated as SDT-induced autophagy as well as the initial autophagic flux blockage by 3-MA. In addition, there was negligible organ damage or inflammatory lesions observed in the H&E staining images of main organs assay (Additional file 1: Figure S13) and no significant changes in blood indexes (Additional file 1: Figure S14, 15) compared to those in the control group after different treatments, confirming the high biosafety and low systemic toxicity during the antitumor treatment. All these results demonstrate that the inhibition of autophagy in combination with SDT could significantly suppress tumor growth without damaging the surrounding normal tissues. Considering the high conceptual advance and convincing synergistic antitumor effect of SDT and autophagy inhibition, the strategy to appropriately regulate autophagic processes can be extended to other cancer-therapeutic modalities, such as photodynamic therapy or chemotherapy, based on confirming in advance that these therapeutic modalities will cause cytoprotective autophagy.

## Conclusions

In summary, we initially demonstrated that nanosonosensitizers-medicated SDT induced cytoprotective autophagy attributing to enhanced production of ROS in breast cancer cells, and explored the expression profiles of mRNAs in MCF-7 cells treated with SDT by emoloying high-throughput RNA sequencing. Further transcriptomic and bioinformatics analysis of these sequencing results revealed that the predicted genes were mainly involved in MAPK and AMPK signaling pathways associated with autophagy. Especially, cancer cells were prone to acquire resistance to SDT due to the pro-survival role of autophagy. Based on the specific mechanism of inhibiting pro-survival autophagy, we successfully proposed an intriguing combined therapeutic strategy integrated with SDT and autophagy blockage for achieving high antitumor efficacy based on biocompatible nanoliposomes composed of both sonosensitizer (PpIX) and autophagy inhibitor (3-MA). It has been systematically demonstrated that inhibition of autophagy in cancer cells remarkably decreased the cell resistance to intracellular oxidative stress and reinforced SDT effect, inducing superb tumor-suppression outcome in a biosafe way. Therefore, the intracellular generated ROS by PpIX component in nanosonosensitizer disrupted cellular homoeostasis, drove cancer cells to become acutely dependent on autophagy, thereby enhancing its vulnerability and response to 3-MA mediated autophagy inhibition. This strategy on simultaneously combing SDT and autophagy blockage represents an efficient paradigm for the treatment of ROS-resistant cancer by autophagy inhibition-augmented SDT.

## Supplementary Information

**Additional file 1**: **Figure S1**. Zeta potential of Lip, PpIX@Lip and PpIX/3-MA@Lip. Values are presented as means ± s.d. (n=3) *P < 0.05. **Figure S2**. Continuous measurements of hydrodynamic size and Zeta potential of nanoliposomes. **Figure S3**. High pressure liquid chromatograph (HPLC) analysis and the encapsulation efficiency and loading capacity of drugs. **Figure S4**. Time dependent DPBF absorption spectra of PpIX/3-MA@Lip nanoliposome. **Figure S5**. Protein quantitative analysis of LC3B, p62, and c-PARP. **Figure S6**. Expression profiling changes of mRNAs in control group and SDT group. **Figure S7**. GO analyses of differentially expressed mRNAs as induced by SDT. **Figure S8**. KEGG signaling pathway analyses of differentially expressed mRNAs as induced by SDT. **Figure S9**. Time-dependent cellular uptake analysis of nanoliposomes; **Figure S10**. Quantitative analysis of ROS-generating capability. **Figure S11**. Cell viability of MCF-7 gradually decreased in a dose-dependent pattern. **Figure S12**. IVFIS analysis. **Figure S13**. H&E-stained tissue sections of major organs. **Figure S14 and 15**. Hematological biochemical examination for in vivo biosafety evaluation.

## Data Availability

The data are available in the main manuscript, supplementary Information files, and from the corresponding authors upon reasonable request.

## Abbreviations

PTT: Photothermal therapy
US: Ultrasound
ROS: Reactive oxygen species
^1^O_2_: Singlet oxygen
SL: Sonoluminescence
PpIX: Protoporphyrin IX
3-MA: 3-Methyladenine
PI3K: Phosphoinositide 3-kinase
MAPK: Mitogen-activated protein kinase
AMPK: Adenosine 5 ‘-monophosphate (AMP)-activated protein kinase
NAC: N-acetylcysteine
CCK-8: Cell-counting kit-8
TEM: Transmission electron microscopy
HPLC: High-performance liquid chromatography
DLS: Dynamic light scattering
ESR: Electron paramagnetic resonance
LIFU: Low-intensity focused ultrasound
MMP: Mitochondrial membrane potential
CLSM: Confocal laser scanning microscopy
LC3: Light chain 3
NAC: N-acetylcysteine
GO: Gene Ontology
KEGG: Kyoto Encyclopedia of Genes and Genomes
GSEA: Gene set enrichment analysis
ATGs: Autophagy-related genes
IRS1: Insulin receptor substrate 1
PE: Phosphatidyleth- anolamine
IVFIS: In vivo Fluorescence imaging system
H&E: Haematoxylin–eosin
TUNEL: TdT-mediated dUTP nickend labelling
