# Supplementary material for: Autophagy blockade synergistically enhances nanosonosensitizer-enabled sonodynamic cancer nanotherapeutics
Source: J Nanobiotechnology. 2021 Apr 20;19:112. doi: 10.1186/s12951-021-00855-y (PMC8056542; doi:10.1186/s12951-021-00855-y)
Supplement: Supplementary file 1 — Additional file 1: Figure S1. Zeta potential of Lip, PpIX@Lip and PpIX/3-MA@Lip. Values are presented as means ± s.d. (n=3) *P < 0.05. Figure S2. Continuous measurements of hydrodynamic size and Zeta potential of nanoliposomes. Figure S3. High pressure liquid chromatograph (HPLC) analysis and the encapsulation efficiency and loading capacity of drugs. Figure S4. Time dependent DPBF absorption spectra of PpIX/3-MA@Lip nanoliposome. Figure S5. Protein quantitative analysis of LC3B, p62, and c-PARP. Figure S6. Expression profiling changes of mRNAs in control group and SDT group. Figure S7. GO analyses of differentially expressed mRNAs as induced by SDT. Figure S8. KEGG signaling pathway analyses of differentially expressed mRNAs as induced by SDT. Figure S9. Time-dependent cellular uptake analysis of nanoliposomes; Figure S10. Quantitative analysis of ROS-generating capability. Figure S11. Cell viability of MCF-7 gradually decreased in a dose-dependent pattern. Figure S12. IVFIS analysis. Figure S13. H&E-stained tissue sections of major organs. Figure S14 and 15. Hematological biochemical examination for in vivo biosafety evaluation. [file 12951_2021_855_MOESM1_ESM.doc]

Supporting information for

**Autophagy Blockade Synergistically Enhances Nanosonosensitizer-Enabled Sonodynamic Cancer Nanotherapeutics**

Liqiang Zhou^†^, Minfeng Huo^‖^, Xiaoqin Qian^¶^, Li Ding^‖^,*, Luodan Yu^∮^, Wei Feng^∮^, XinwuCui^†^,* and Yu Chen^‖,∮^,*

^†^Sino-German Tongji-Caritas Research Center of Ultrasound in Medicine, Department of Medical Ultrasound, Tongji Hospital, Tongji Medical College, Huazhong University of Science and Technology, Wuhan, 430030, P. R. China. E-mail: cuixinwu@hust.edu.cn (X. W. Cui)

^∮^School of Life Sciences Shanghai University, Shanghai, 200444, P. R China. E-mail: chenyuedu@shu.edu.cn (Y. Chen)

^‖^State Key Laboratory of High Performance Ceramics and Superfine Microstructure, Shanghai Institute of Ceramics, Chinese Academy of Sciences, Shanghai, 200050, P. R. China. E-mail: dlgood2006@mail.sic.ac.cn (L. Ding)

^¶^Department of Ultrasound, Affiliated People's Hospital of Jiangsu University, Zhenjiang 212002, P, R. China.

**
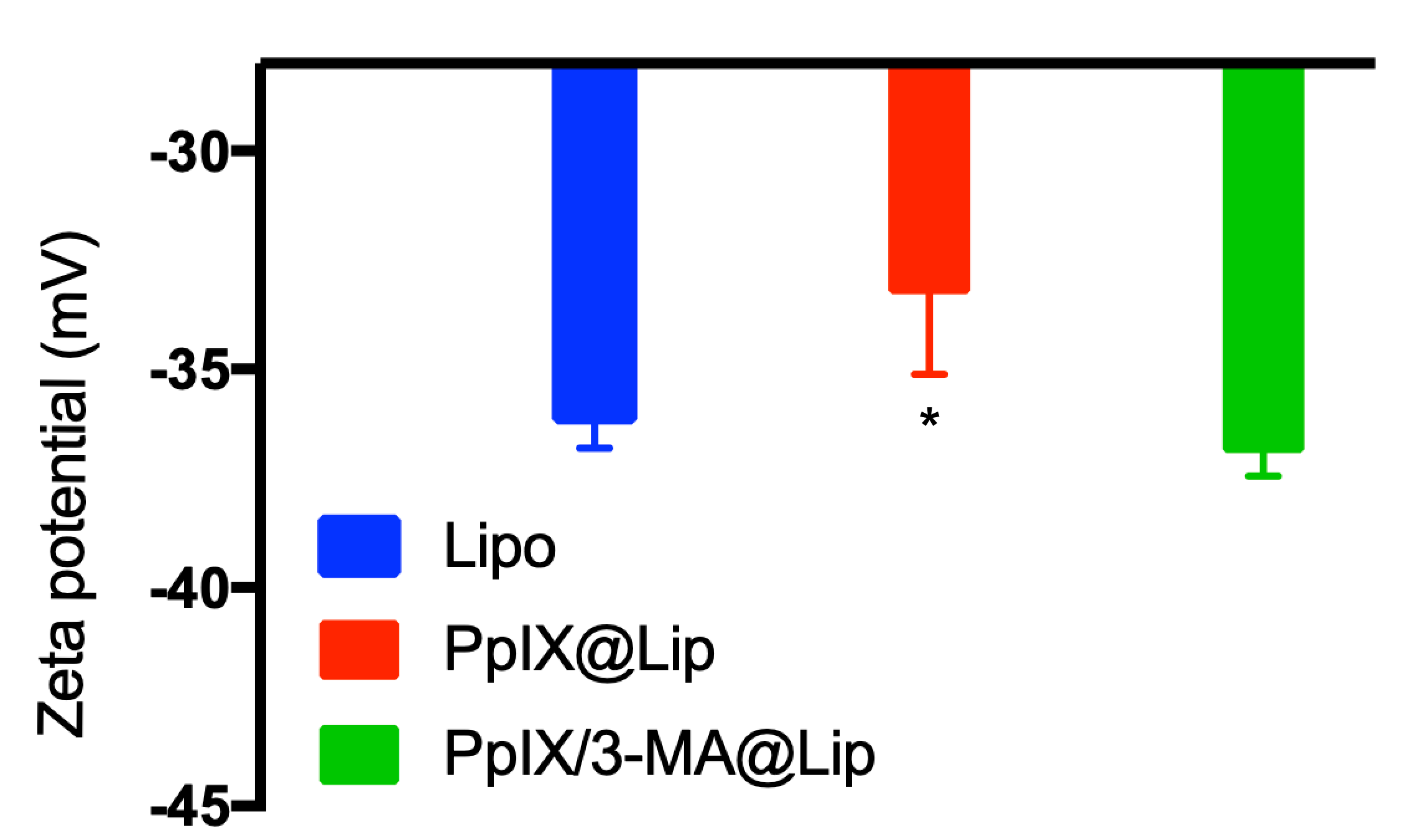
**

**Figure S1.** Zeta potential of Lip, PpIX@Lip and PpIX/3-MA@Lip. Values are presented as means ± s.d. (n=3) **P* < 0.05.


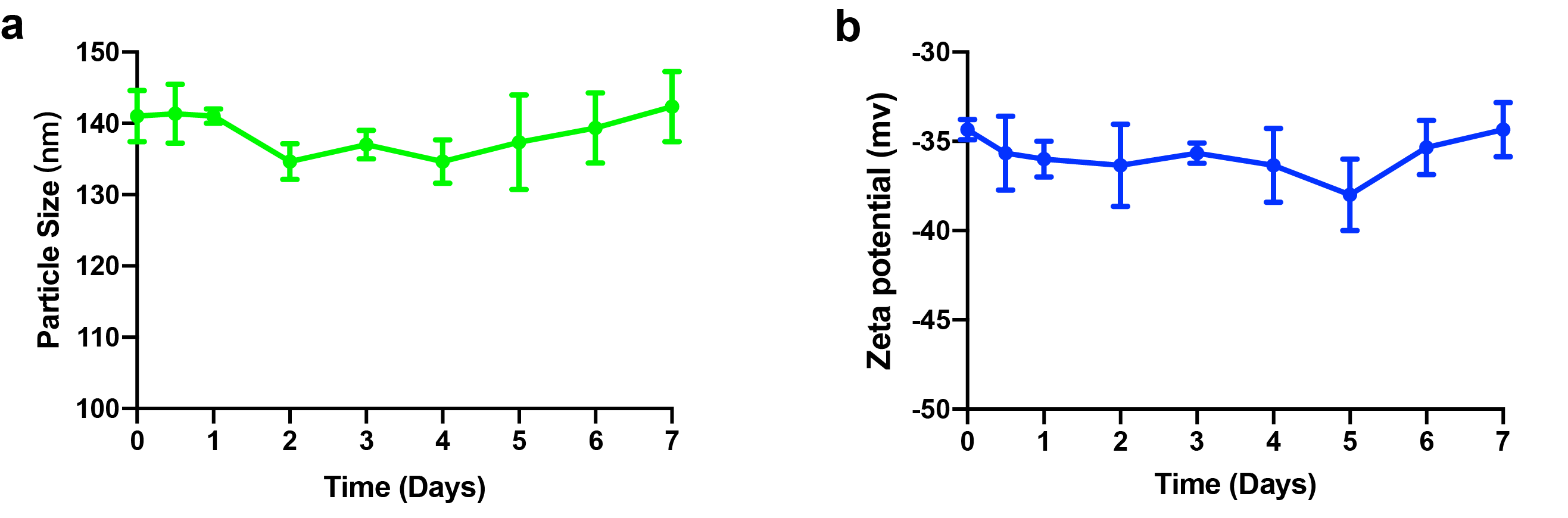


**Figure S2.** Time-dependent particle size and Zeta potential changes of drug-loaded nanoliposmes in PBS at 4 °C. Quantitative data are presented as means ± s.d. (n = 3).

**
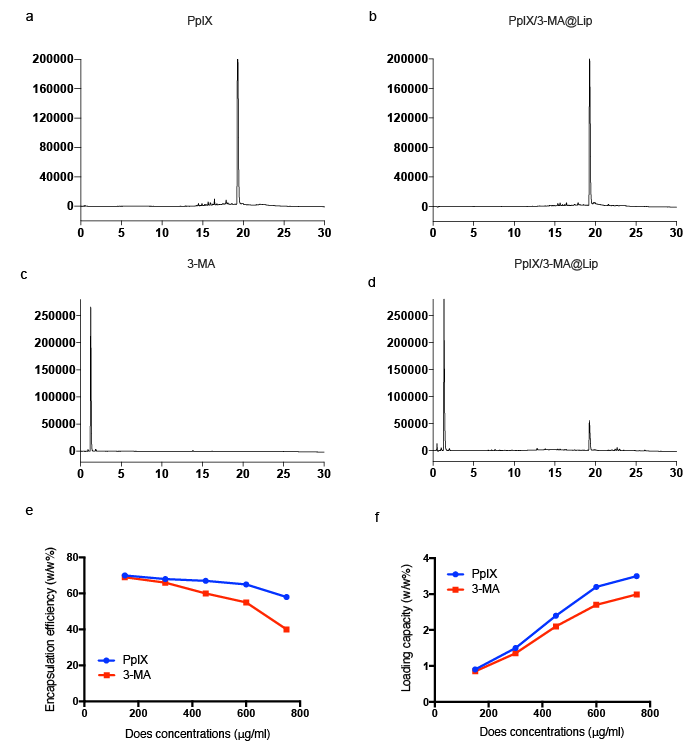
**

**Figure S3.** High pressure liquid chromatograph (HPLC) absorbance of (a) PpIX, (c) 3-MA and (b, d) PpIX/3-MA@Lip. The similar absorbance peak of PpIX and PpIX/3-MA@Lip, and 3-MA and PpIX/3-MA@Lip indicates the successful co-loading of PpIX and 3-MA into nanoliposomes; (e, f) The encapsulation efficiency (e) and loading capacity (f) of PpIX and 3-MA by nanoliposomes obtained at different dose concentrations.

**Figure S4.** Time dependent DPBF absorption spectra of PpIX/3-MA@Lip nanoliposome at the wavelength of 410 nm in UV−vis spectrum under US irradiation (LIFU, 1.0 MHz, 1.5 W cm^-2^, 1 min).


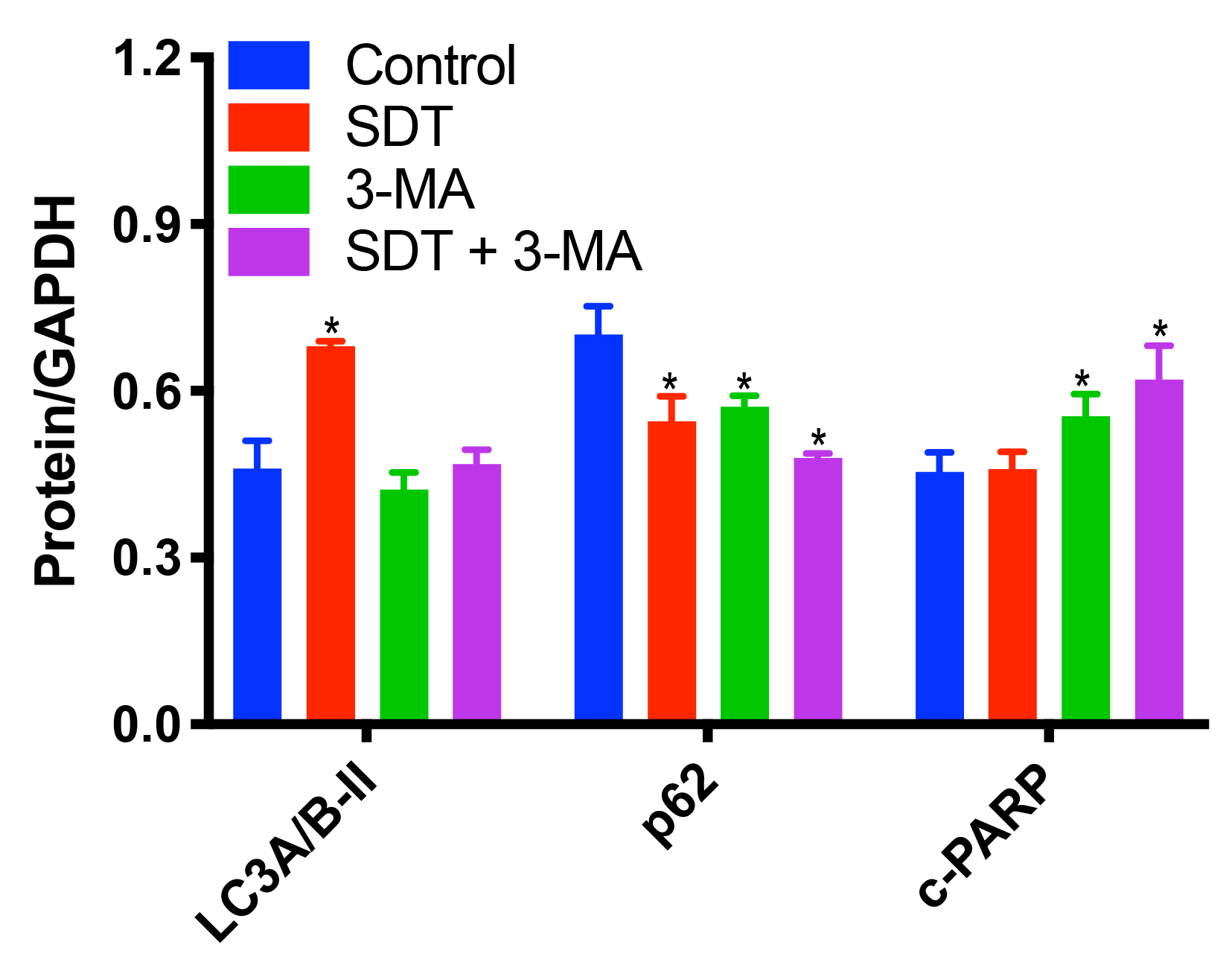


**Figure S5.** Protein quantitative analysis of LC3B, p62, and c-PARP expressions in MCF-7 cells after different treatments for 24 h. GAPDH expression levels serve as the loading controls. Quantitative data are presented as means ± s.d. (n = 3) **P* < 0.05.


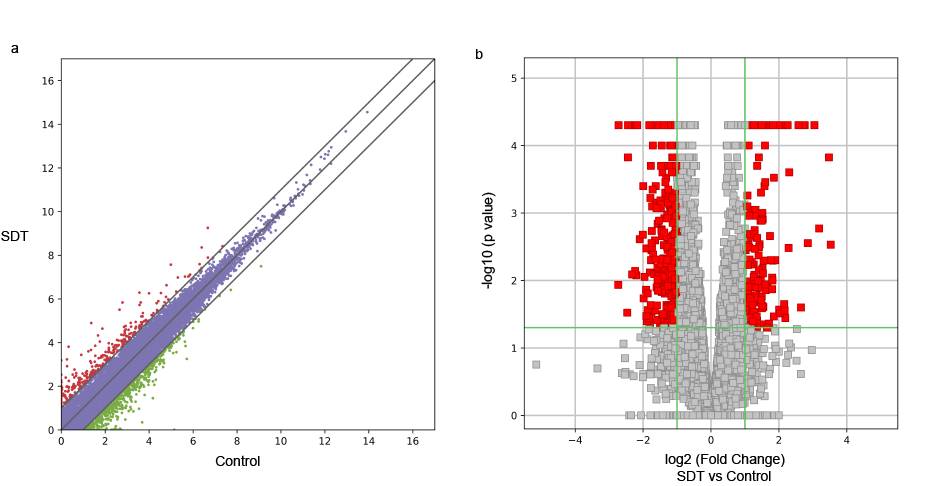


**Figure S6.** Expression profiling changes of mRNAs in control group and SDT group. (a) Scatter plot and (b) volcano plot indicating upregulated and downregulated mRNAs in SDT group when compared with control group.


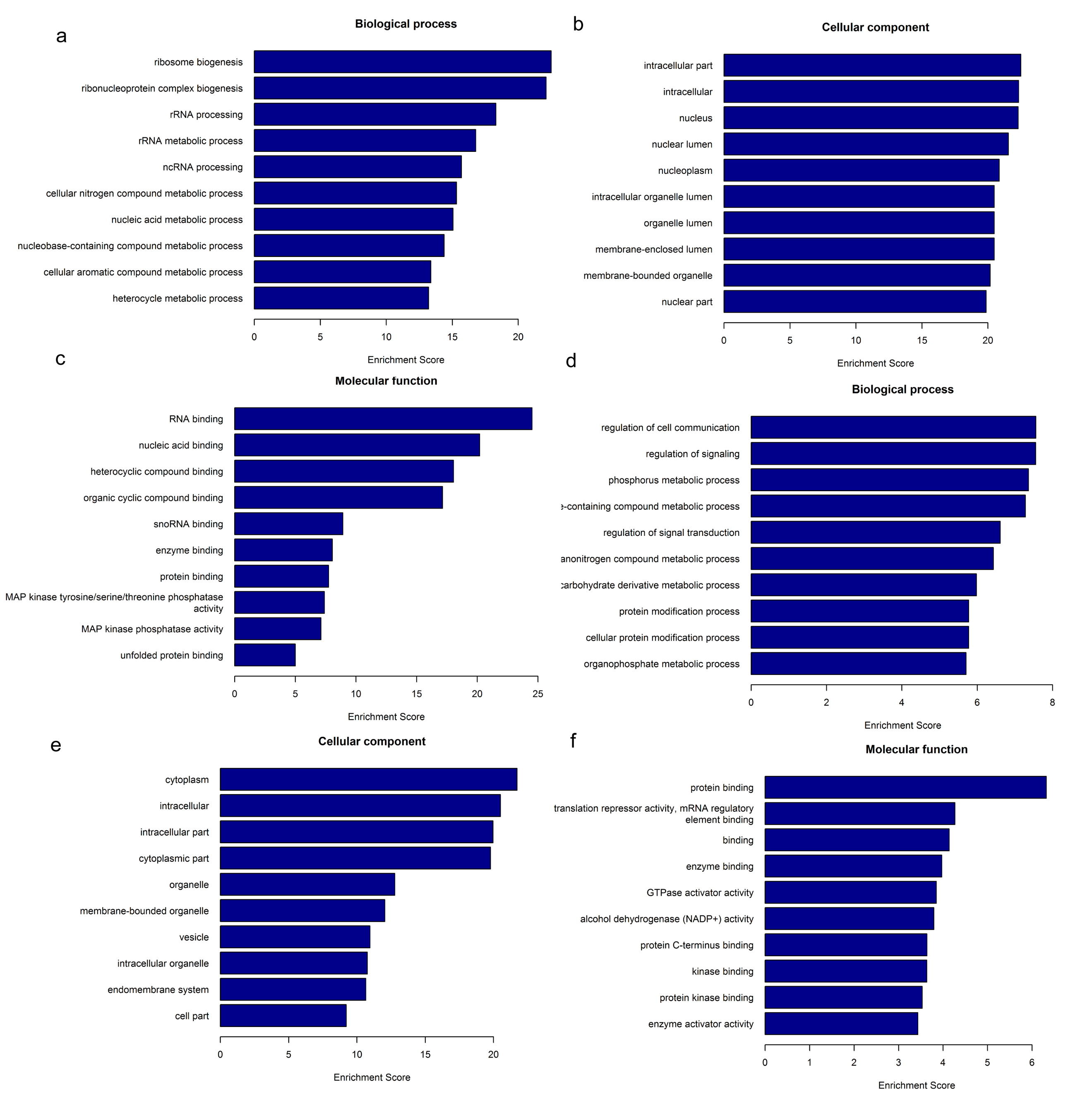


**Figure S7.** GO analyses of differentially expressed mRNAs as induced by SDT. (a) Biological process (BP), (b) cellular component (CC), and (c) molecular function (MF) of upregulated mRNAs. (d) BP, (e) CC, and (f) MF of downregulated mRNAs.


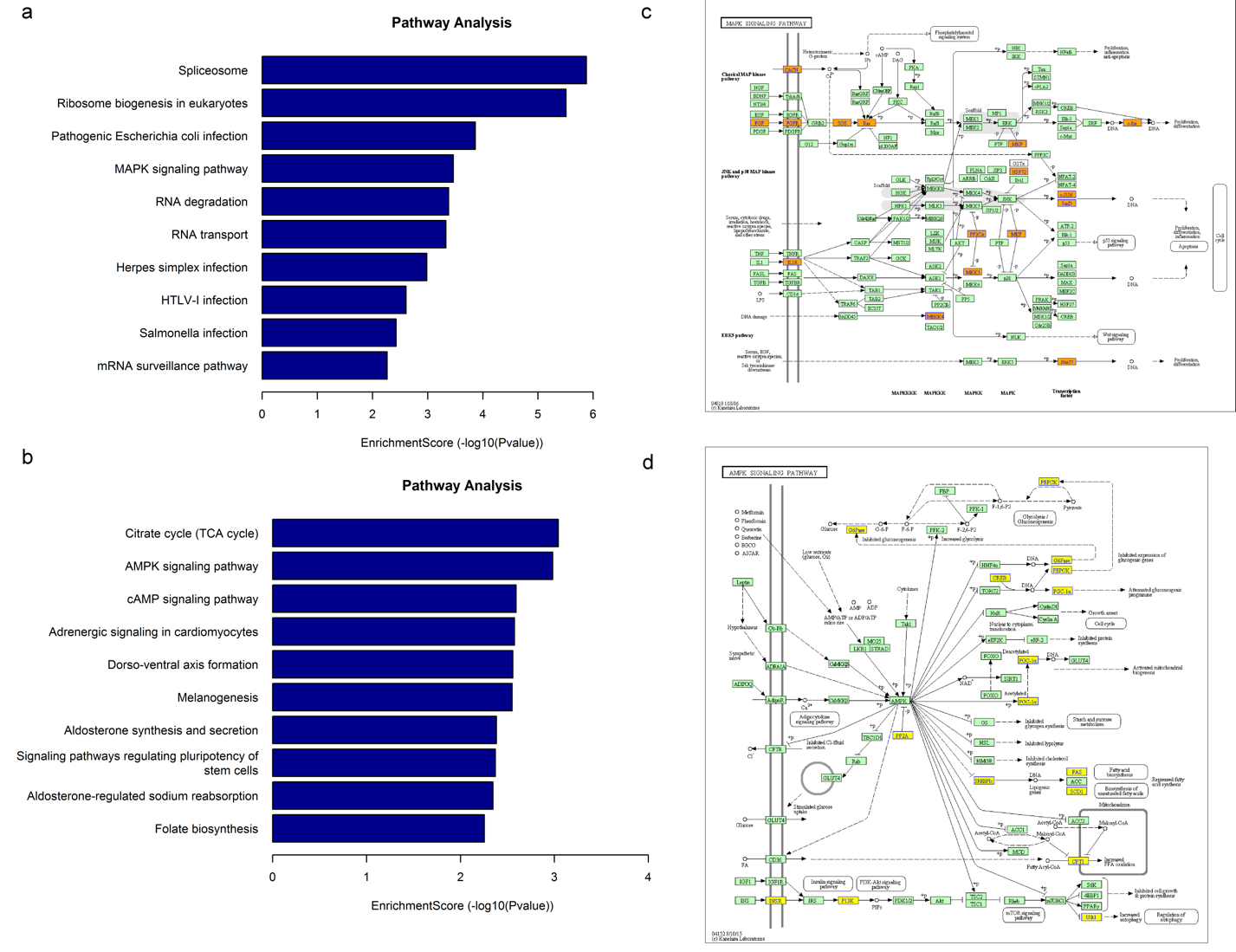


**Figure S8.** KEGG signaling pathway analyses of differentially expressed mRNAs as induced by SDT. The bar plot shows the top 10 enrichment score [–log10 (*P* value)] of the significantly enriched pathways for (a) upregulated mRNAs and (b) downregulated mRNAs. (c) and (d) exhibits upregulated and downregulated genes in MAPK and AMPK signaling pathways.


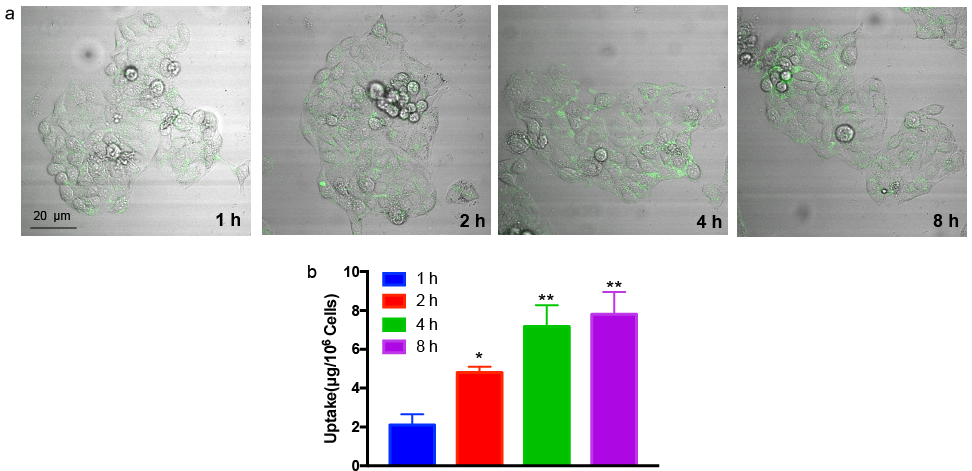


**Figure S9.** (a) CLSM images showing the time-dependent cellular uptake of PpIX/3-MA@Lip in MCF-7 cells. Scale bar = 20 μm; (b) Time-dependent quantitative analysis of cellular uptake of PpIX/3-MA@Lip in MCF-7 cells; Quantitative data are presented as means ± s.d. (n = 3) **P* < 0.05, ***P* < 0.01.

**
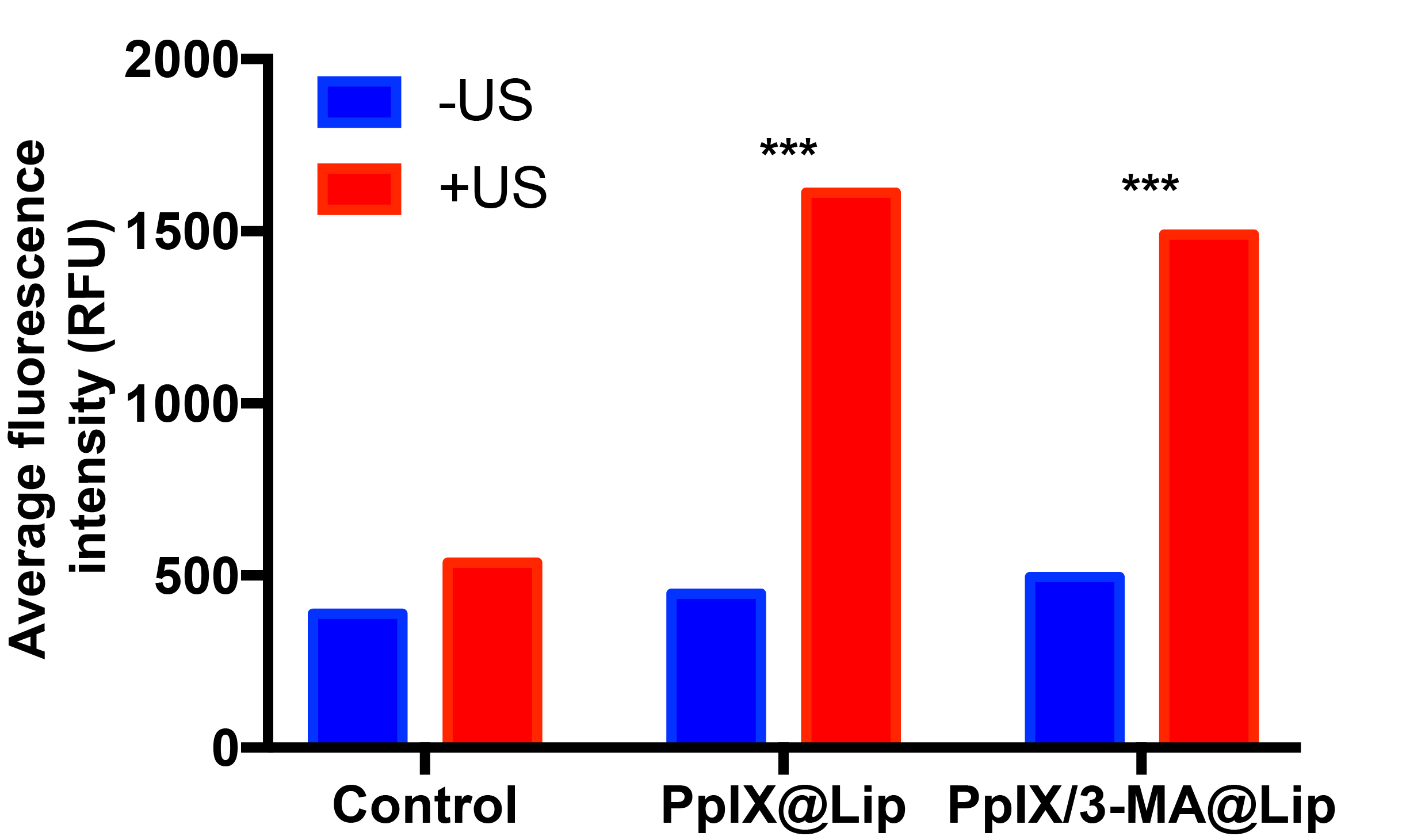
**

**Figure S10.** Average fluorescence intensity profiles at the ROI in CLSM images stained with DCFH-DA to quantitatively analyze ROS-generating capability of nanoliposomes under US irradiation (LIFU, 1.0 MHz, 1.5 W cm^-2^, 1min). Quantitative data are presented as means ± s.d. (n = 3) ****P* < 0.001. Data was obtained using Olympus software.


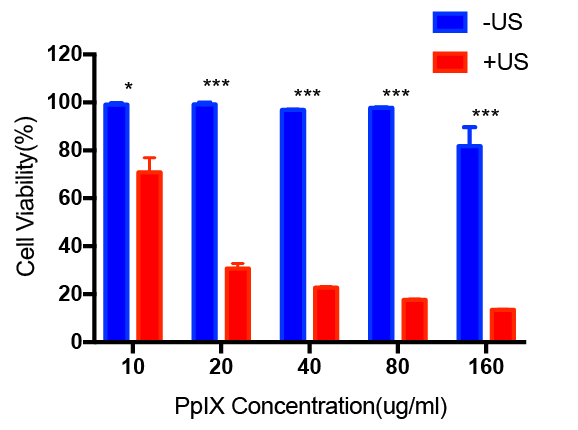


**Figure S11.** Cell viability of MCF-7 gradually decreased in a dose-dependent pattern. Cell viabilities of MCF-7 cells after co-incubation with PpIX/3-MA@Lip for 24 h and exposed to US irradiation at varied concentrations (LIFU, 1.0 MHz, 1.5 W cm^−2^, 1 min, 50% duty cycle). Values are presented as means ± s.d. (n = 3) **P* < 0.05, ****P* < 0.001.


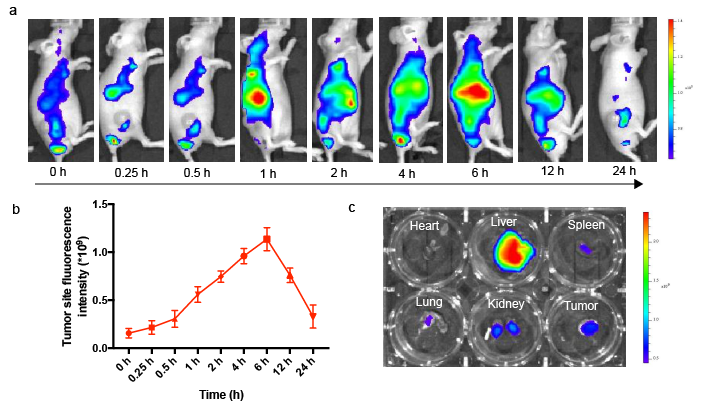


**Figure S12.** (a, b) *In* *vivo* biodistribution (a) and fluorescence intensity (b) profiles of Cy5.5-conjugated nanoliposomes into tumor-bearing nude mice at predetermined time points (0 h, 0.25 h, 0.5 h, 1 h, 2 h, 4 h, 6 h, 12 h, and 24 h). (c) Fluorescence images of dissected major organs and tumors from nude mice for *in* *vivo* biodistribution experiment.


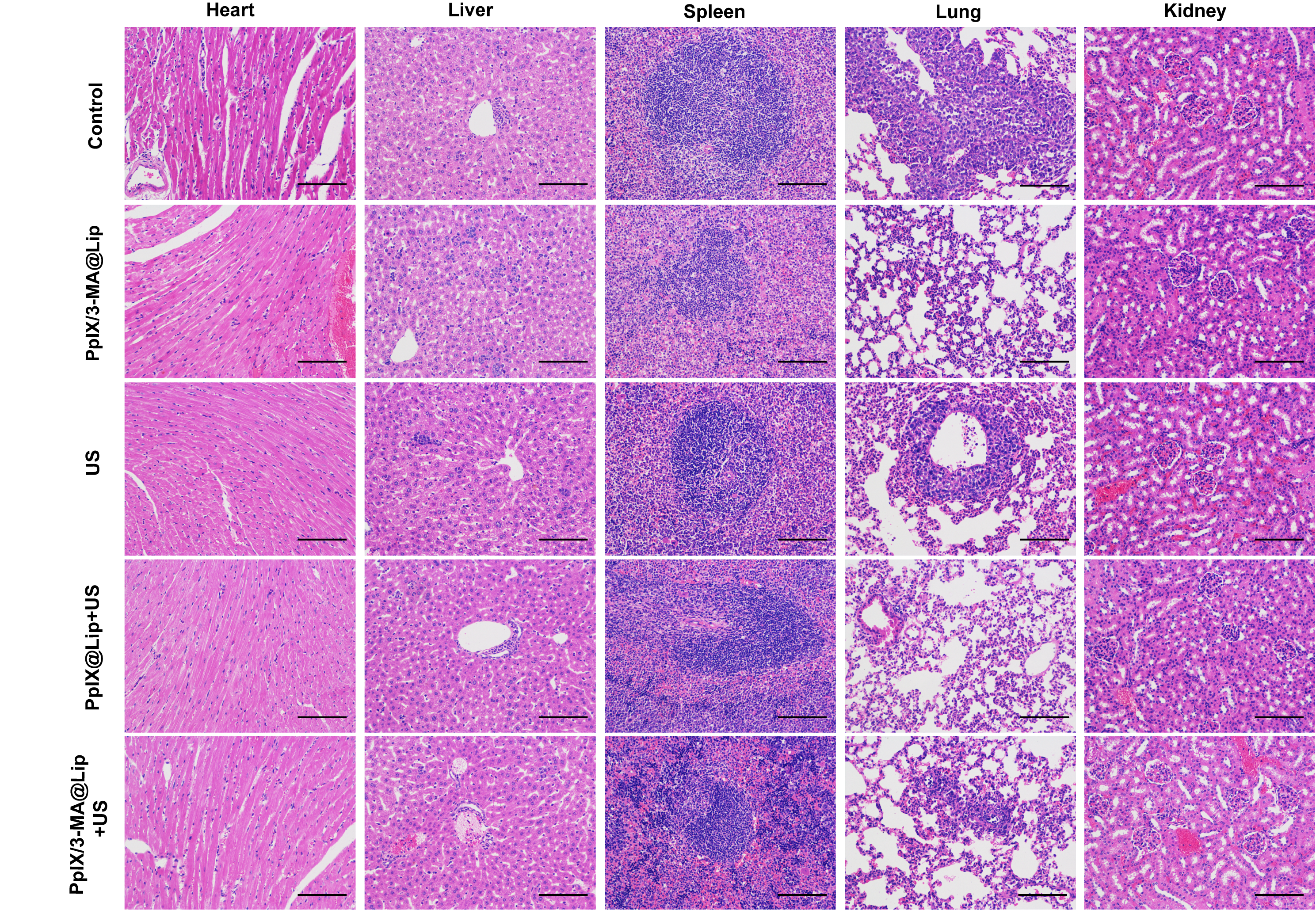


**Figure S13.** H&E-stained tissue sections of major organs (heart, liver, spleen, lung and kidney) from mice with different treatments. All scale bar = 100 μm.

**Figure S14.** Hematological biochemical indexes of healthy female Kunming mice with different treatments were evaluated for *in vivo* biosafety conditions after 28 d (n = 5 in each groups). Values are presented as means ± s.d.

**
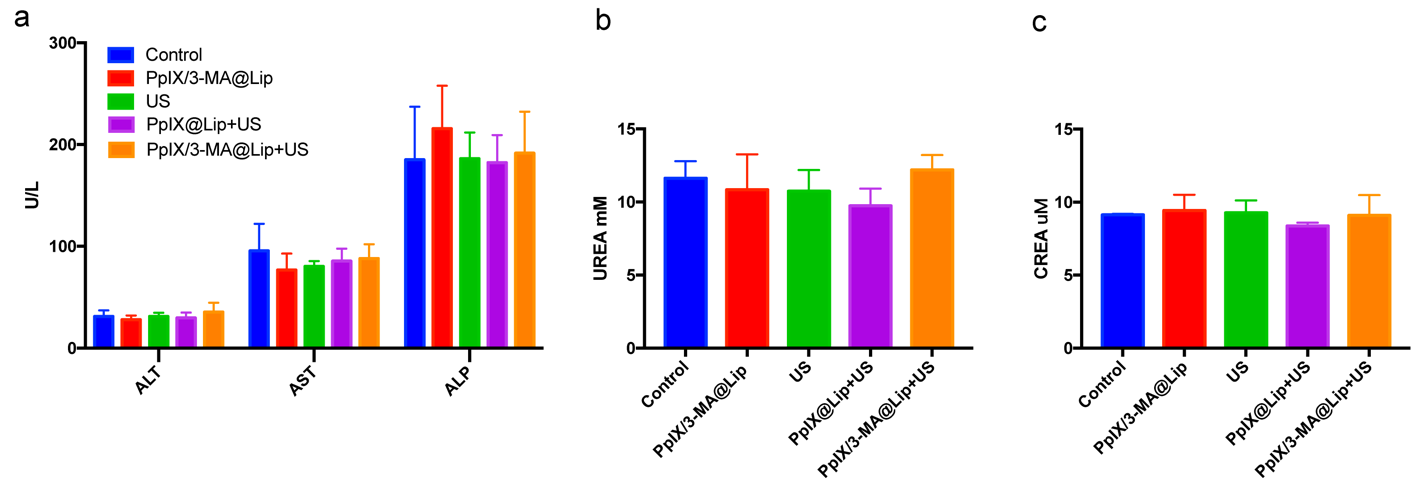
**

**Figure S15.** (a) Hepatic and (b, c) renal biochemical indexes of healthy female Kunming mice with different treatments were evaluated for *in vivo* biosafety conditions after 28 d (n = 5 in each groups). Values are presented as means ± s.d.
